# Cultivation, Genetic, Ethnopharmacology, Phytochemistry and Pharmacology of *Moringa oleifera* Leaves: An Overview

**DOI:** 10.3390/ijms160612791

**Published:** 2015-06-05

**Authors:** Alessandro Leone, Alberto Spada, Alberto Battezzati, Alberto Schiraldi, Junior Aristil, Simona Bertoli

**Affiliations:** 1 International Center for the Assessment of Nutritional Status (ICANS), University of Milan, Via Sandro Botticelli 21, 20133 Milan, Italy; E-Mails: alberto.battezzati@unimi.it (A.B.); simona.bertoli@unimi.it (S.B.); 2 Department of Food, Environmental and Nutritional Sciences (DeFENS), University of Milan, Via Mangiagalli 25, 20133 Milan, Italy; E-Mail: alberto.schiraldi@unimi.it; 3 Department of Agricultural and Environmental Sciences-Production, Landscape, Agroenergy (DISAA), University of Milan, Via Celoria 2, 20133 Milan, Italy; E-Mails: alberto.spada@unimi.it (A.S.); junior.aristil@unimi.it (J.A.)

**Keywords:** *Moringa oleifera*, ethnopharmacology, phytochemistry, pharmacology, diabetes, dislipidemia, cancer, genetic variability, molecular markers, breeding

## Abstract

*Moringa oleifera* is an interesting plant for its use in bioactive compounds. In this manuscript, we review studies concerning the cultivation and production of moringa along with genetic diversity among different accessions and populations. Different methods of propagation, establishment and cultivation are discussed. *Moringa oleifera* shows diversity in many characters and extensive morphological variability, which may provide a resource for its improvement. Great genetic variability is present in the natural and cultivated accessions, but no collection of cultivated and wild accessions currently exists. A germplasm bank encompassing the genetic variability present in Moringa is needed to perform breeding programmes and develop elite varieties adapted to local conditions. Alimentary and medicinal uses of moringa are reviewed, alongside the production of biodiesel. Finally, being that the leaves are the most used part of the plant, their contents in terms of bioactive compounds and their pharmacological properties are discussed. Many studies conducted on cell lines and animals seem concordant in their support for these properties. However, there are still too few studies on humans to recommend Moringa leaves as medication in the prevention or treatment of diseases. Therefore, further studies on humans are recommended.

## 1. Origin and Geographical Distribution

**Figure 1:**
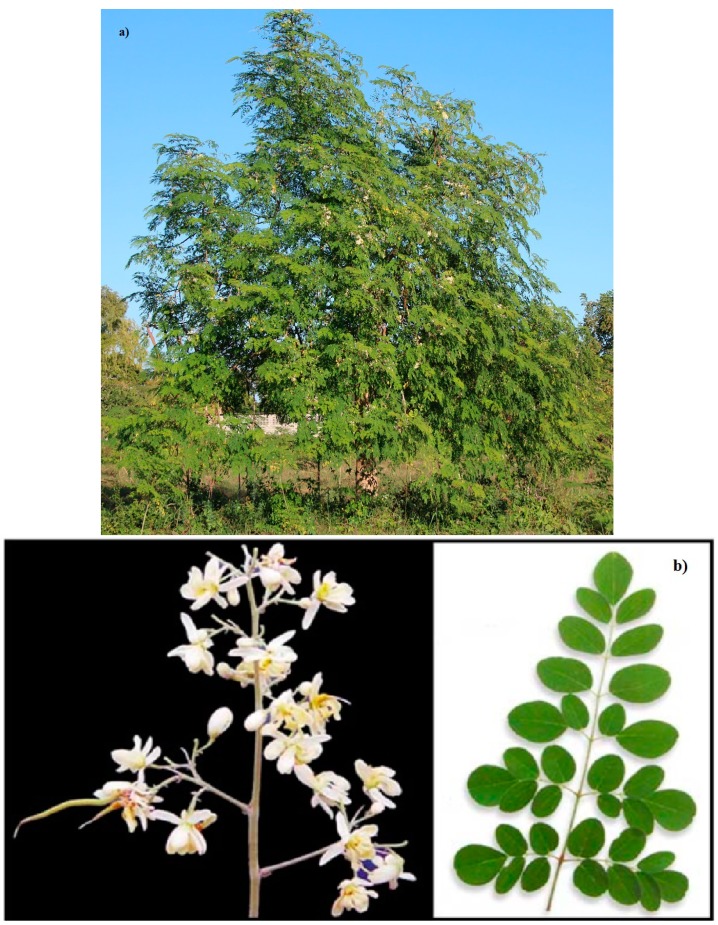
(**a**) A tree of *Moringa oleifera*; (**b**) Moringa flowers and leaves.

In the monogeneric genus Moringa of Moringaceae family there are 13 species (namely, *M. arborea*, indigenous to Kenya; *M. rivae* indigenous to Kenya and Ethiopia; *M. borziana*, indigenous to Somalia and Kenia; *M. pygmaea* indigenous to Somalia; *M. longituba* indigenous to Kenia, Ethiopia and Somalia; *M. stenopetala* indigenous to Kenya and Ethiopia; *M ruspoliana* indigenous to Ethiopia; *M. ovalifolia* indigenous to Namibia and Angola; *M. drouhardii*, *M. hildebrandi* indigenous to Madagascar; *M. peregrine* indigenous o Red sea and Horn of Africa, *M. concanensis*, *Moringa oleifera* indigenous to sub-Himalayan tracts of Northern India [1]), among which *Moringa oleifera* (Figure 1) has so far become the most used and studied.

This species is a fast growing soft wood tree that can reach 12 m in height and is indigenous to the Himalayan foothills (northern India Pakistan and Nepal) [2,3]. Its multiple uses and potential attracted the attention of farmers and researchers in past historical eras. Ayurvedic traditional medicine says that *Moringa oleifera* can prevent 300 diseases and its leaves have been exploited both for preventive and curative purposes [4]. Moreover, a study in the Virudhunagar district of Tamil Nadu India reports Moringa among the species utilized by traditional Siddha healers [5]. Ancient Egyptians used *Moringa oleifera* oil for its cosmetic value and skin preparation [6]; even if the species never became popular among Greeks and Romans, they were aware of its medical properties [7]. *Moringa oleifera* has been grown and consumed in its original areas until recently (the 1990s) when a few researchers started to study its potential use in clarifying water treatments, while only later were its nutritional and medical properties “discovered” and the species was spread throughout almost all tropical countries. In 2001, the first international conference on *Moringa oleifera* was held in Tanzania and since then the number of congresses and studies increased disseminating the information about the incredible properties of *Moringa oleifera*. Now this species has been dubbed “miracle tree”, or “natural gift”, or “mother’s best friend”.

*Moringa oleifera* grows in any tropical and subtropical country with peculiar environmental features, namely, dry to moist tropical or subtropical clime, with annual precipitation of 760 to 2500 mm (it requires less than 800 mm irrigation) and temperature between 18 and 28 °C. It grows in any soil type, but heavy clay and waterlogged, with pH between 4.5 and 8, at an altitude up to 2000 m [8,9].

A study on local uses and geographical distribution of *Moringa oleifera* [10] that covers the major agro-ecological region in Nigeria, clearly established that “though considered a not indigenous species, *Moringa oleifera* has found wide acceptance among various ethnic Nigeria, who have exploited different uses (e.g., food, medicine, fodder *etc.*).

Nowadays, *Moringa oleifera* and its derivatives are distributed mainly in Middle East, African and Asian countries [11] and are still spreading to other areas.

## 2. Cultivation and Production

*Moringa oleifera* development is achieved in two main ways: sowing and cutting.

Traditionally in Sudan the seeds are preferred while vegetative propagation is common in India, Indonesia and in some areas of West Africa [8].

Sowing requires selection of the seeds, when they are easily available and human labor is limited, while the possibility to transplant seedlings allows flexibility in field planting even if it requires extra labor and costs.

Seeds germinate within two weeks, at a maximum 2 cm depth. When sowing is planned in nursery, the seedlings can be transplanted when they reach about 30 cm (3–6 weeks after germination) [12].

The number of seeds per kilogram ranges from 3000 to 9000, depending on the variety, with a germination rate of 80%–90% for ideal storage conditions (3 °C, 5%–8% moisture). However, the viability decreases if seeds remain at ambient temperature and high relative humidity, their germination rate dropping to 7.5% after three months [3,13].

Cutting is preferred when seeds availability is scarce and/or when labor is not a limiting factor. Ramachandran *et al.* [14] reports that plants raised from seeds produce fruits of poorer quality, while Animashaun *et al.* [15] suggest that trees grown from seeds develop longer roots (an advantage for stabilization and access to water) compare to that grown from cuttings that have much shorter roots.

When hard woodcuttings (1–2 m long 4–16 cm diameter [8,15]) from adult trees are planted during the rainy season burying one third in the soil, they readily develop roots that in few months reach a considerable size [16]. *Moringa oleifera* is an exceptionally fast growing tree, in three months it can be 3 m high and in few years reaches 12 m if it is left to growth naturally. Since the tree vigorously re-sprouts after cutting, pruning or pollarding are usually practiced to enhance lateral branching and give the tree a bush shape in order to facilitate the harvest. Nevertheless, since literature reports about the good practice management of *Moringa oleifera* are scant, practical trials are needed [12]. Leaves and seeds are the parts of the plant of interest. Accordingly, the spatial distribution in planting *Moringa oleifera* trees is designed to facilitate the relevant harvest and the management practices.

For production of leaves, *Moringa oleifera* plantation can be designed as follows:

(i) intensive production with spacing ranging from 10 cm × 10 cm to 20 cm × 20 cm, harvest interval between 35 to 45 days, irrigation and fertilization are needed;
(ii) semintensive production with spacing about 50 cm × 100 cm, harvest interval between 50 to 60 days, irrigation and fertilization suggested;
(iii) integrate in an agroforestry system with spacing distance of 2–4 m between rows, harvest interval around 60 days, fertilization and irrigation not strictly necessary.

Production decreases from intensive production to less dense spacing (agroforestry system), although a tremendous variability can be observed for a given spatial distribution and the same cultivation management. For example, the yield of an intensive plantation can range from 580 to 40 m/ha/year [15], being season dependent with the largest yield in wet or cold season. There is a need for further studies to assess optimum spacing and harvest intervals that comply with the different climates and production systems [17,18,19]. Harvest can be mechanical or manual. Shoots are cut at a 0.5–1 m height above the ground; but leaves can be picked directly off the tree; this practice, however, albeit quicker, leads to a less vigorous re-growth.

For the production of seed a low density plantation has a positive effect on yields: typically 2.5 × 2.5 m or 3 × 3 m triangular pattern [20]. Fruits (trilobite capsule), referred as pods (brown color and dry and split longitudinally), ripen about three months after flowering and must be harvested as soon as possible. Each pod usually contains about 26 1-cm diameter seeds lined by three whitish papery leaflets on the edge. Like for leaves, also the production of seed shows a tremendous variability. A single tree can produce from 15.000 to 25.000 seeds with an average weight of 0.3 gr per seed [21]; moreover early flowering varieties produce pods in six month, while other varieties require more than one year. After pruning, branches develop new pods within 6 months [1].

## 3. Genetic and Breeding

The major *Moringa oleifera* producer is India with an annual production of 1.1 to 1.3 million tons of tender fruits from an area of 380 km^2^ [22]. Information about the production in other countries is scarce. The great interest in *Moringa oleifera* does not concern its commercial value, being mainly related to its multipurpose uses and its ability to guarantee a reliable yield, while other crops cannot, in countries where people are mostly at risk of suffering from nutritional deficiencies. Indeed its cultivation is localized in developing countries where different parts of the plant are utilized: seeds for oil and water purification; leaves, seeds and fruits for their high nutritional value (nutritional integrator); leaves and seeds for biomass and animal feeding; different parts in traditional medicine. Moreover, Moringa has been planted around the world and is naturalized in many areas (*i.e.*, almost the entire tropical belt) increasing the variability of the species.

As Moringa is a cross-pollinated tree, high heterogeneity in form and yield is expected. Several works indeed report variability in flowering time [23] (from annual type to perennial type), tree nature (from deciduous to evergreen), tree shape (from semi spread to upright), resistance to hairy caterpillar [22,23,24], flowering time (*i.e.*, some tree flowering throughout the year while others flower in two distinct season) [14].

Although *Moringa oleifera* shows diversification in many characters and high morphological variability, which may become a resource for its improvement, the major factors that limit productivity are the absence of elite varieties adapted to local conditions and the use of seeds obtained through open pollination from plants in the planted area. Furthermore, despite the various uses of *Moringa oleifera* and its morphological differentiation, the number of accessions to collections and active germplasm banks are incipient across the world.

Many ecotypes are present in India: Jaffna (soft and taste fruits), Chavakacheri murungai (similar to Jaffna), Chemmurungai (red tipped fruits), Kadumurungai (small and inferior fruits) Palmurungai (bitter taste), Punamurungai (similar to Palmurungai), Kodikalmurungai (short fruit), Palmurungai, Puna Murungai and Kodikkal Murungai and wild Kadumurunga [14,25]. Recently two varieties (PKM-1; PKM2) have been developed at Horti Nursery Networks, Tamil Nadu, India, to improve pod production: usually those varieties are grown as annual; after two harvests the tree is dragged out and new seedlings are planted [26]. At Kerala Agricultural University (India) several varieties have been developed and available.

Outside India there are research centers focused on *Moringa oleifera* improvement across the world: AVDRC (Taiwan), Rural development initiative (Zambia), Moringa Philippines foundation (Philippines) Moringa community (Zambia).

In spite of the great variability of *Moringa oleifera* no institutions have a germplasm bank or data base with either cultivated or spontaneous accessions. The divergence between genetic variability inherent to the species and poor variability reflected in germplasm banks should be fixed since it represents an obstacle for the progress of breeding programs.

Moringa cytological studies revealed that *Moringa oleifera* has 2c genome size of 1.2 pg [27] and it is a true diploid with 2*n* = 28 [14]. Since 1999 molecular markers have become standards for the genetic characterization of *Moringa oleifera*. Studies started with the use of dominant markers until the development of co-dominant markers (in 2010) that allow distinction between homozygotes and heterozygotes, which provides optimal genetic information profiles.

Interestingly, out of 2857 scientific publications on *Moringa oleifera* in the primary database (Web of Science), only 12 include genetic characterization based on molecular markers. Furthermore, only 77 fragments of DNA and RNA sequences are available (data from NCBI nucleotide database). This means that the genetic approach and its potential application in breeding programs are just at the beginning step. The common aim of almost all the studies is the genetic diversification among different populations and/or accessions: dominant markers are the most used (66% of all papers).

In spite of the limited range of dominant markers (heterozygote cannot be distinguished from homozygote specimens), the studies among commercial, cultivated or natural accessions have contributed to the understanding of genetic variability of *Moringa oleifera*. In this context, Amplified Fragment Length Polymorphism (AFLP) and Random Amplified Polymorphic DNA (RAPD) analyses along with Inter-Simple Sequence Repeat (ISSR) and cytochrome P_450_ were used. Muluvi *et al.* [28] used AFLP to investigate seven natural populations from India and introduced populations in Malawi and Kenya. Authors found a significant level of population differentiation and separation of genotypes based on geographical origin. Moreover, high portion of genetic variability was within Indian accessions. In line with these findings, the authors argued that Kenya populations presumably came from India, as suggested by the small number of genetically related accessions. Thank to the same molecular markers [29] the outcrossing rate in *Moringa oleifera* was detected: 26% of selfing in *Moringa* trees. This evidence had a strong impact on the breeding program, as inbreed lines and hybridization allowed improvement of the species.

RAPD were used by different authors to investigate cultivated and non-cultivated population of Tanzania [30], different accessions in Nigeria [31], accessions present in Embra Cosatal Teblelands Sergipe germplasm bank in Brazil [32], commercially grown varieties in India [33], new genotype developed in different countries (Thailand, USA, India and Malaysia, Tanzania, Taiwan) [34], and further accessions in Nigeria [35]. All these studies showed the higher level of genetic diversity in natural population with respect to the cultivated ones. Cultivated accessions present in the considered germplasm banks are genetically close and need to be widen to promote increased diversity and used in breeding programs. Many studies disagree with Muluvi’s conclusions that a significant level of population differentiation and separation of genotypes can be based on the geographical origin. Indeed no clusters were found according to geographical origins. This could be due to the planting spread that produced a high rate of gene flow through cross-pollination. Interestingly, Popoola *et al.* [35] investigated morpho-metric characters along with molecular markers and showed a good correlation between 100 seeds weight with pod length, pod weight, number of seeds per lobule and number of seed per pod.

Studies on *Moringa oleifera* with co-dominat markers started in 2010 when Wu *et al.* [36] developed microsatellite markers. The first successful estimates of genetic diversity and population structure were obtained with Simple Sequence Repeat (SSR) in 2013 by Shahazad *et al.* [37]. These authors evaluated accessions collected in different locations of Pakistan and accessions from different countries (India, Tanzania, Senegal, Mozambique, Zimbabwe, Florida, Mexico, Haiti, Belize) obtained from Educational Concerns foe Hunger Organization (ECHO). They found high genetic diversity in wild Pakistan accessions, whereas low genetic diversity in ECHO accessions. Moreover, ECHO accessions are more similar to those of a single province of Pakistan. Most likely, British colonialists introduced *Moringa oleifera* in early of twentieth century in Africa from India, while in the 1784 an Englishman took *Moringa oleifera* over to Jamaica [37]. The export pathway was restricted to Indian coastal region (where most movement of goods and people took place) and involved a relatively small number of accessions that belonged to a common or few populations. This explains the low genetic variability within ECHO accessions with respect to the Pakistan ones.

Later on, a further investigation on twelve Indian populations, from northern and southern regions of India, was performed through SSR together with morphological markers [38]. In this study too, individuals from various geographical areas were not significantly different genetically, while a large variability exist in Indian populations. Morphological analysis on fourteen quantitative and eleven qualitative characters showed correlation among some quantitative characters, e.g., between tree tallness with fruit girth, trunk girth with tree branching. More SSR were identified in 2014 thanks to EST examination involving several plant species [39] and not utilized so far in *Moringa oleifera* genetic investigation.

Even if all the reported studies are valuables and have a tremendous importance for conservation, selection and collection of *Moringa oleifera* seeds, same questions are still to be addresses in order to develop an improved *Moringa oleifera* cultivation. Considering the cultivation challenges, some research activities should be prioritized: (i) collection and characterization of world accessions both cultivated and natural in order to obtain a true understanding of the genetic diversity and structure of *Moringa oleifera*; (ii) set a collaborative network among National and International Research Centre, O.N.G, farmers that already work on *Moringa oleifera.*

This will help scientists and producers to:

- have a reliable access to information about genetics and materials to develop better *Moringa* varieties and technologies for farming practices: phenotypic characterization is a priority to evaluated the accessions;
- ensure that Moringa production is improved along with best cultivation practice;
- focus research on the association between phenotypic and molecular data within the contest of breeding;
- define maps (both association map and physical map) to identify genes that may confer resistance to biotic and abiotic stress and quantitative traits loci (QTL) for a possible introgression of genes into commercial and cultivated accessions.

Today next generation sequencing (NGS) [40] is an approachable tool to discovery genome-wide genetic markers. This technique could be applied to species with no existing genome data like Moringa. Thank to NGS a saturated genetic map could be obtain within reasonable cost and time, in turn interesting characters could be identified and exploited in breeding programmes.

## 4. Traditional Uses

All plant parts of *Moringa oleifera* are traditionally used for different purposes, but leaves are generally the most used [10,41]. In particular, they are used in human and animal nutrition and in the traditional medicine. Leaves are rich in protein, mineral, beta-carotene and antioxidant compounds, which are often lacking among the populations of underdeveloped or developing countries. *Moringa* leaves are added to food preparations as integrators of the diet. In traditional medicine, these leaves are used to treat several ailments including malaria, typhoid fever, parasitic diseases, arthritis, swellings, cuts, diseases of the skin, genito-urinary ailments, hypertension and diabetes. They are also used to elicit lactation and boost the immune system (to treat HIV/AIDS related symptoms) [10,41,42,43,44,45], as well as cardiac stimulants and contraceptive remedy. One can directly consume either raw and dried leaves or the extract of an aqueous infusion.

Similarly, the use of seeds concerns both human nutrition and traditional medicine. Barks are boiled in water and soaked in alcohol to obtained drinks and infusions that can be used to treat stomach ailments (ease stomach pain, ulcer and aiding digestion), poor vision, joint pain, diabetes, anemia and hypertension [10,43], toothache, hemorrhoids, uterine disorder [10,44]. In a well known practice, *Moringa* seeds are used to sediment impurities of water [10].

Roots are soaked in water or alcohol and boiled with other herbs to obtained drinks and infusions as remedies for toothache, as anthelmintic and antiparalytic [10,41,42] drugs and as sex enhancers.

Finally, flowers are used to produce aphrodisiac substances and to treat inflammations, muscle diseases, hysteria, tumors and enlargement of the spleen [42,44].

## 5. Non Food or Medicinal Uses

Beyond the uses of Moringa as a food and for human health, other possible uses exist. It can be used as a natural plant growth enhancer; indeed leaves are rich in zeatin (a plant hormone belong to the cytokinin group). Leaf extracts can stimulate plant growth and increasing crop yield. Researches performed using a spray based on leaf extracts of wheat, maize and rice support the wide range of beneficial effect on crops [46].

Moringa seed powder can be used for water purification, replacing dangerous and expensive chemicals such as aluminum sulfate [10].

Interestingly, leaf extracts and also seed extracts show biopesticide activity, effective against larvae and adults of *Trigoderma granarium* and can reduce the incidence of fungi on groundnut seeds [46].

One of the interesting applications of Moringa seeds is their utilization as biomass for biodiesel production.

Due to the increasing energy demand and environmental problems associated with fossil fuels, the improvement of alternative fuels and renewable sources of energy is required. Biodiesel can replace petroleum-derived oil (petrodiesel), without any sulphur or aromatic compound and with lower emission of monoxides, hydrocarbons and particulates. Furthermore, biodiesel can reduce dependence on imported fuels: a crucial problem in developing countries [47].

Moringa seeds have an oil content of 30%–40%, with a high-quality fatty acid composition *i.e.*, high oleic acid (>70%) [48]. In addition they posses significant resistance to oxidative degradation. These proprieties make Moringa oil a good candidate to produce biodiesel after transesterificaton [48,49,50]. Biswas and John [51], in a study conducted in Australia, report that approximately 3030 kg of oil are required to produce 1000 liters of biodiesel. Furthermore, an equivalent of 3.03 tonnes/ha of oil seeds can be harvested from dry land, and 6.06 tonnes/ha can be harvested from irrigated land. Since biodiesel production with Moringa seed oil is a second generation production (*i.e.*, not in direct competition with existing farmland and with food crops) and as Moringa can grown on degraded land, studies suggest that Moringa biodiesel is an acceptable substitute to fossil fuels, even when compared against biodiesel derived from vegetable oil of other species.

## 6. Phytochemistry

As *Moringa oleifera* leaves are most used part of the plant, we review articles concerning phytochemistry and pharmacological properties of leaves.

**Figure 2:**
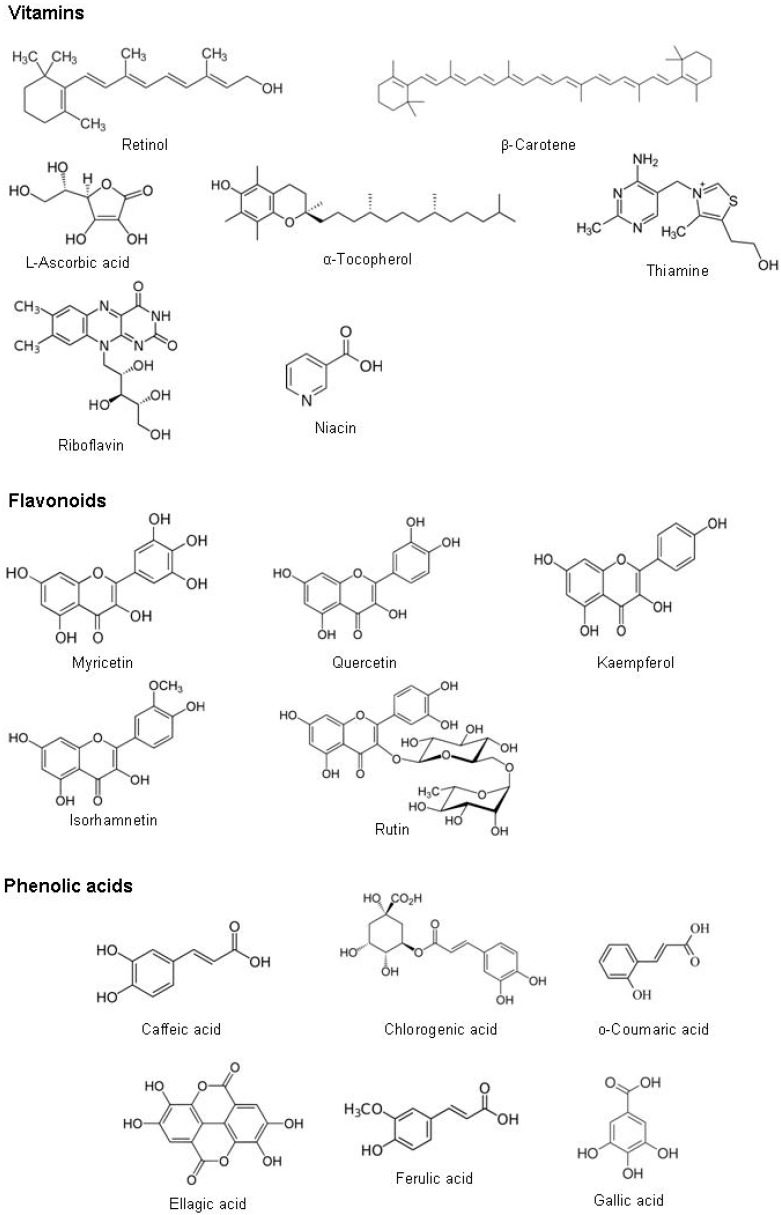

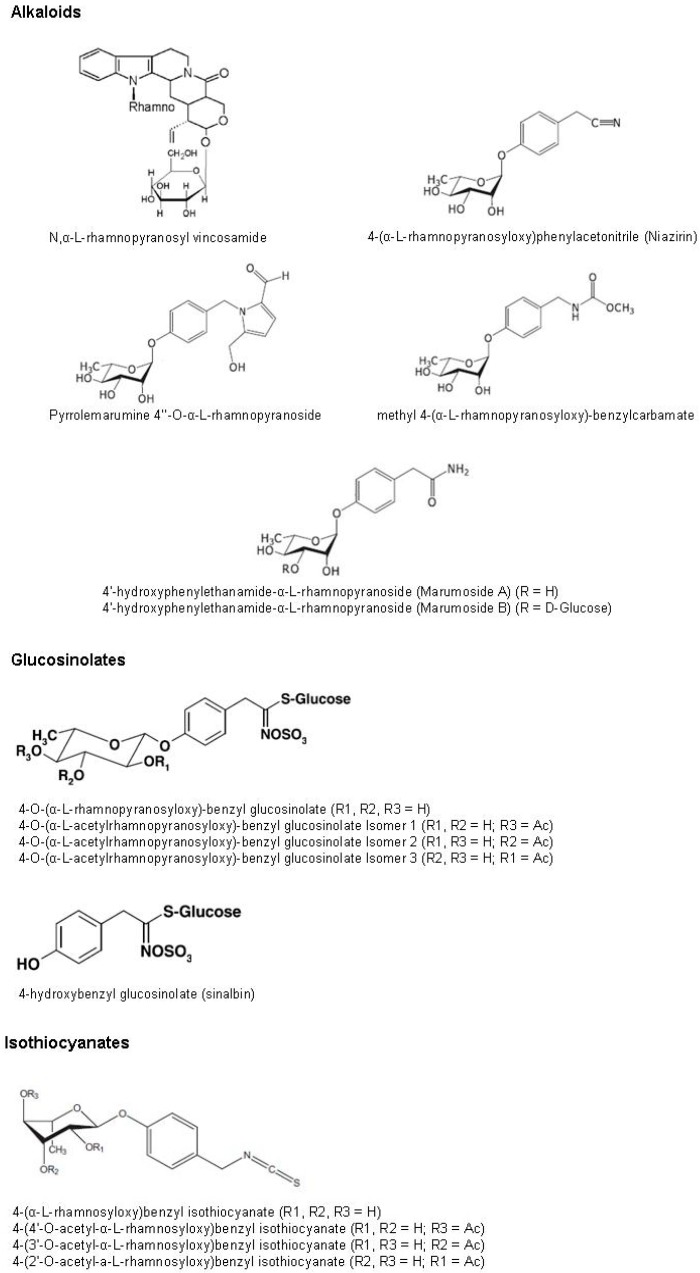
Chemical structure of bioactive compounds found in *Moringa oleifera* leaves.

Several bioactive compounds were recognized in the leaves of *Moringa oleifera*. They are grouped as vitamins, carotenoids, polyphenol, phenolic acids, flavonoids, alkaloids, glucosinolates, isothiocyanates, tannins, saponins and oxalates and phytates (Figure 2). The amounts of different bioactive compounds found in *Moringa oleifera* leaves and reported in literature are summarized in Table 1, Table 2, Table 3, Table 4, Table 5, Table 6, Table 7, Table 8 and Table 9.

**Table 1:** Vitamins content in *Moringa oleifera* leaves.

Bioactive Compound	Leaves	Value Found in Literature	Value Express as Dry Weight	Drying Method	Extractive Method	Analytical Method	Country	Reference
**Vitamins**								
Vitamin A	fresh	11,300 IU	45,200 IU		N/A	N/A	India	[14]
	fresh	23,000 IU	92,000 IU ^a^		N/A	N/A	Brazil	[52]
Vitamin B1–Thiamine	fresh	0.06 mg/100 g	0.24 mg/100 g		N/A	N/A	India	[14]
	fresh	0.21 mg/100 g	0.84 mg/100 g		N/A	N/A	N/A	[53]
	fresh	0.6 mg/100 g	2.58 mg/100 g		N/A	Microbiological method	India	[54]
	dried	2.64 mg/100 g	2.85 mg/100 g	N/A	N/A	N/A	N/A	[53]
Vitamin B2–Riboflavin	fresh	0.05 mg/100 g	0.20 mg/100 g		N/A	N/A	India	[14]
	fresh	0.05 mg/100 g	0.20 mg/100 g		N/A	N/A	N/A	[53]
	fresh	0.17 mg/100 g	0.726 mg/100 g		N/A	Microbiological method	India	[54]
	dried	20.5 mg/100 g	22.16 mg/100 g	N/A	N/A	N/A	N/A	[53]
Vitamin B3–Niacin	fresh	0.8 mg/100 g	3.20 mg/100 g		N/A	N/A	India	[14]
	fresh	0.8 mg/100 g	3.20 mg/100 g		N/A	N/A	N/A	[53]
	fresh	0.82 mg/100 g	3.5 mg/100 g		N/A	Microbiological method	India	[54]
	dried	8.2 mg/100 g	8.86 mg/100 g	N/A	N/A	N/A	N/A	[53]
Vitamin C–Ascorbic acid	fresh	220 mg/100 g	880 mg/100 g		N/A	N/A	India	[14]
	dried	17.3 mg/100 g	18.7 mg/100 g	N/A	N/A	N/A	N/A	[53]
	dried	92 mg/100 g	92 mg/100 g	Sun-drying for 4 days	N/A	AOAC 2004	India	[55]
		140 mg/100 g	140 mg/100 g	Shadow-drying for 6 days				
		56 mg/100 g	56 mg/100 g	Oven-drying at 60 °C for 1 h				
	dried	38.8 mg/100 g ^b^	38.8 mg/100 g ^b^	Air-drying	Metaphosphoric acid	Indophenol titration	Pakistan	[56]
	freeze-dried	271 mg/100 g	271 mg/100 g	Freeze-drying	Deionized water	Colorimetric method	Florida, USA	[57]
	freeze-dried	920 mg/100 g	920 mg/100 g	Freeze-drying	6% metaphosphoric acid	Titration against 2,6-dichlorophenolindophenol	Nicaragua	[58]
		840 mg/100 g	840 mg/100 g				India	
		680 mg/100 g	680 mg/100 g				Niger	
Vitamin E–Tocopherol	fresh	9.0 mg/100 g	16.21 mg/100 g		N-hexane + ethyl acetate + BHT	Reverse-phase HPLC	Malaysia	[59]
	dried	113 mg/100 g	122.16 mg/100 g	N/A	N/A	N/A	N/A	[53]
	dried	74.45 mg/100 g	74.45 mg/100 g	Drying at 60 °C for 8 h	Microscale saponification and extraction with n-hexane	HPLC	Mexico	[60]
	dried	77.0 mg/100 g	85.08 mg/100 g	Air-dried under shade	N/A	HPLC Fluorescence	South Africa	[61]

Abbreviations: ^a^ Obtained considering a moisture of 75%; ^b^ Mean value of samples collected in different seasons; N/A = Not available.

**Table 2:** Carotenoids content in *Moringa oleifera* leaves.

Bioactive Compound	Leaves	Value Found in Literature	Value Express as Dry Weight	Drying Method	Extractive Method	Analytical Method	Country	Reference
**Carotenoids**								
β-carotene	fresh	6.63 mg/100 g	33.48 mg/100 g		Acetone–n-hexane	HPLC	Taiwan	[62]
	fresh	6.8 mg/100 g	27.22 mg/100 g		N/A	N/A	N/A	[53]
	dried	36 mg/100 g	36 mg/100 g	Sun-drying for 4 days	N/A	AOAC 2004	India	[55]
		39.6 mg/100 g	39.6 mg/100 g	Shadow-drying for 6 days				
		37.8 mg/100 g	37.8 mg/100 g	Oven-drying at 60 °C for 1 h				
	dried	16.3 mg/100 g	17.62 mg/100 g	N/A	N/A	N/A	N/A	[53]
	dried	18.5 mg/100 g	20.44 mg/100 g	Air-dried under shade	N/A	HPLC	South Africa	[61]
	freeze-dried	66 mg/100 g	66 mg/100 g	Freeze-drying	Acetone	HPLC	Florida, USA	[57]
Lutein	fresh	6.94 mg/100 g	35.05 mg/100 g		Acetone–n-hexane	HPLC	Taiwan	[62]
	freeze-dried	102 mg/100 g	102 mg/100 g	Freeze-drying	Acetone	HPLC	Florida, USA	[57]

Abbreviation: N/A = Not available.

**Table 3:** Polyphenols content in *Moringa oleifera* leaves.

Bioactive Compound	Leaves	Value Found in Literature	Value Express as Dry Weight	Drying Method	Extractive Method		Analytical Method	Country	Reference
**Polyphenols**									
Total phenols	dried	4581 mgGAE/100 g ^a^	4581 mgGAE/100 g ^a^	Shade-drying	Water Soxhlet extraction for 18–20 h		Folin-Ciocalteau	India	[63]
		3602 mgGAE/100 g ^b^	3602 mgGAE/100 g ^b^						
	dried	3290 mgGAE/100 g	3290 mgGAE/100 g	N/A	50% MeOH		Folin-Ciocalteau	India	[64]
	dried	2090 mgGAE/100 g	2090 mgGAE/100 g	N/A	50% MeOH, 100% MeOH and water		Folin-Ciocalteau	India	[65]
	dried	10,504 mgGAE/100 g	10,504 mgGAE/100 g	N/A	Water at 80 °C for 2 h		Folin-Ciocalteau	India	[66]
	dried	10,616 mgGAE/100 g ^c^	10,616 mgGAE/100 g ^c^	Air-drying	80% MeOH		Folin-Ciocalteau	Pakistan	[56]
	dried	10,300 mgGAE/100 g	10,300 mgGAE/100 g	Air-drying	100% MeOH	Extraction by shaker	Folin-Ciocalteau	Pakistan	[67]
		12,200 mgGAE/100 g	12,200 mgGAE/100 g		80% MeOH				
		9720 mgGAE/100 g	9720 mgGAE/100 g		100% EtOH				
		11,600 mgGAE/100 g	11,600 mgGAE/100 g		80% EtOH				
	dried	9630 mgGAE/100 g	9630 mgGAE/100 g	Air-drying	100% MeOH	Extraction by reflux	Folin-Ciocalteau	Pakistan	[67]
		10,700 mgGAE/100 g	10,700 mgGAE/100 g		80% MeOH				
		6160 mgGAE/100 g	6160 mgGAE/100 g		100% EtOH				
		8210 mgGAE/100 g	8210 mgGAE/100 g		80% EtOH				
	dried	2070 mg TAE/100 g	2070 mg TAE/100 g	Air-drying	Acetone/Water (7:3)		Folin-Ciocalteau	India	[68]
	dried	1600 mgTEA/100 g ^d^	1600 mgTEA/100 g ^d^	Air-drying	80% EtOH		Folin-Ciocalteau	Nicaragua	[69]
		3400 mgTEA/100 g ^e^	3400 mgTEA/100 g ^e^						
	dried	5350 mgCAE/100 g	5350 mgCAE/100 g	Oven-drying at 60 °C for 24 h	Maceration with 70% EtOH		Folin-Ciocalteau	Thailand	[70]
		2930 mgCAE/100 g	2930 mgCAE/100 g		Maceration with 50% EtOH				
		3710 mgCAE/100 g	3710 mgCAE/100 g		Percolation with 70% EtOH				
		3280 mgCAE/100 g	3280 mgCAE/100 g		Percolation with 50% EtOH				
		4550 mgCAE/100 g	4550 mgCAE/100 g		Soxhlet extraction with 70% EtOH				
		4460 mgCAE/100 g	4460 mgCAE/100 g		Soxhlet extraction with 50% EtOH				
	freeze-dried	1535.6 mgGAE/100 g	1535.6 mgGAE/100 g	Freeze-drying	80% EtOH		Folin-Ciocalteau	Florida, USA	[57]

Abbreviations: ^a^ Mature/old leaves; ^b^ Tender/young leaves; ^c^ Mean value of samples collected in different seasons; ^d^ Extracted leaves; ^e^ Unextracted leaves; N/A = Not available; GAE = Gallic acid equivalent; TAE = Tannin acid equivalent; CAE = Chlorogenic acid equivalent.

**Table 4:** Phenolic acids content in *Moringa oleifera* leaves.

Bioactive Compound	Leaves	Value Found in Literature	Value Express as Dry Weight	Drying Method	Extractive Method	Analytical Method	Country	Reference
**Phenolic acids**								
Caffeic acid	dried	ND	ND	N/A	50% MeOH	HPLC and MS/MS	India	[64]
	dried	0.409 mg/g	0.409 mg/g	N/A	50% MeOH, 100% MeOH and water	HPLC	India	[65]
	freeze-dried	0.536 mg/g	0.536 mg/g	Freeze-drying	80% EtOH	HPLC	Florida, USA	[57]
Chlorogenic acid	dried	0.018 mg/g	0.018 mg/g	N/A	50% MeOH	HPLC and MS/MS	India	[64]
	dried	0.489 mg/g	0.489 mg/g	N/A	Water at 80 °C for 2 h	HPLC and MS/MS	India	[66]
*o*-Coumaric acid	freeze-dried	6.457 mg/g	6.457 mg/g	Freeze-drying	80% EtOH	HPLC	Florida, USA	[57]
*p*-Coumaric acid	freeze-dried	ND	ND	Freeze-drying	80% EtOH	HPLC	Florida, USA	[57]
Ellagic acid	dried	ND	ND	N/A	50% MeOH, 100% MeOH and water	HPLC	India	[65]
	dried	0.009 mg/g	0.018 mg/g	N/A	50% MeOH	HPLC and MS/MS	India	[64]
	dried	0.189 mg/g	0.189 mg/g	N/A	Water at 80 °C for 2 h	HPLC and MS/MS	India	[66]
Ferulic acid	dried	0.078 mg/g	0.078 mg/g	N/A	50% MeOH	HPLC and MS/MS	India	[64]
	dried	0.078 mg/g	0.078 mg/g	N/A	50% MeOH, 100% MeOH and water	HPLC	India	[65]
	dried	0.128 mg/g	0.128 mg/g	N/A	Water at 80°C for 2 h	HPLC and MS/MS	India	[66]
Gallic acid	dried	ND	ND	N/A	50% MeOH, 100% MeOH and water	HPLC	India	[65]
	dried	1.034 mg/g	1.034 mg/g	N/A	50% MeOH	HPLC and MS/MS	India	[64]
	dried	1.034 mg/g	1.034 mg/g	N/A	Water at 80 °C for 2 h	HPLC and MS/MS	India	[66]
Gentistic acid	freeze-dried	ND	ND	Freeze-drying	80% EtOH	HPLC	Florida, USA	[57]
Sinapic acid	freeze-dried	ND	ND	Freeze-drying	80% EtOH	HPLC	Florida, USA	[57]
Syringic acid	freeze-dried	ND	ND	Freeze-drying	80% EtOH	HPLC	Florida, USA	[57]

Abbreviations: ND = Not detected; N/A = Not available.

**Table 5:** Flavonoids content in *Moringa oleifera* leaves.

Bioactive Compound	Leaves	Value Found in Literature	Value Express as Dry Weight	Drying Method	Extractive Method		Analytical Method	Country	Reference
**Flavonoids**									
Total flavonoids	dried	1.29 mg/g ^a^	5.059 mg/g ^a^	Vacuum-drying	MeOH + HCl + ascorbic acid		HPLC-DAD	Taiwan	[71]
	dried	6.0 mg/g ^a,b^	6.0 mg/g ^a,b^	Air-drying	70% MeOH + 0.1% acetic acid		LC/MS	Ghana	[72]
		7.03 mg/g ^a,b^	7.03 mg/g ^a,b^					Senegal	
		12.16 mg/g ^a,b^	12.16 mg/g ^a,b^					Zambia	
	dried	31.28 mgQE/g	31.28 mgQE/g	N/A	Water at 80 °C for 2 h		HPLC and MS/MS	India	[66]
	dried	27.0 mgQE/g ^c^	27.0 mgQE/g ^c^	Shade-drying	Water Soxhlet extraction for 18–20 h		Colorimetric method	India	[63]
		15.0 mgQE/g ^d^	15.0 mgQE/g ^d^						
	dried	96.12 mg ECE/100g ^e^	96.12 mg ECE/100 g ^e^	Air-drying	80% MeOH		Colorimetric method	Pakistan	[56]
	dried	60.6 mgCE/g	60.6 mgCE/g	Air-drying	100% MeOH	Extraction by shaker	Spectrophotometric method	Pakistan	[67]
		86.6 mgCE/g	86.6 mgCE/g		80% MeOH				
		53.3 mgCE/g	53.3 mgCE/g		100% EtOH				
		62.1 mgCE/g	62.1 mgCE/g		80% EtOH				
	dried	59.0 mgCE/g	59.0 mgCE/g	Air-drying	100% MeOH	Extraction by reflux	Spectrophotometric method	Pakistan	[67]
		72.9 mgCE/g	72.9 mgCE/g		80% MeOH				
		41.9 mgCE/g	41.9 mgCE/g		100% MeOH				
		53.1 mgCE/g	53.1 mgCE/g		80% EtOH				
	dried	25.1 mgIQE/g	25.1 mgIQE/g	Oven-drying at 60° C for 24 h	Maceration with 70% EtOH		Colorimetric method	Thailand	[70]
		12.3 mgIQE/g	12.3 mgIQE/g		Maceration with 50% EtOH				
		18.0 mgIQE/g	18.0 mgIQE/g		Percolation with 70% EtOH				
		14.6 mgIQE/g	14.6 mgIQE/g		Percolation with 50% EtOH				
		24.5 mgIQE/g	24.5 mgIQE/g		Soxhlet extraction with 70% EtOH				
		12.7 mgIQE/g	12.7 mgIQE/g		Soxhlet extraction with 50% EtOH				
	freeze-dried	61.62 mgRE/g	61.62 mgRE/g	Freeze-drying	80% EtOH		Spectrophotometric method	Florida, USA	[57]
	freeze-dried	44.3 mgRE/g	44.3 mgRE/g	Freeze-drying	80% MeOH		Spectrophotometric method	Nicaragua India Niger	[58]
		21.0 mgRE/g	21.0 mgRE/g					Nicaragua India Niger	
		38.1 mgRE/g	38.1 mgRE/g					Nicaragua India Niger	
Apigenin	dried	ND	ND	N/A	MeOH + HCl + ascorbic acid		HPLC	Taiwan	[71]
Daidzein	dried	ND	ND	N/A	50% MeOH, 100% MeOH and water		HPLC	India	[65]
Epicatechin	freeze-dried	5.68 mg/g	5.68 mg/g	Freeze-drying	80% EtOH		HPLC	Florida, USA	[57]
Genistein	dried	ND	ND	N/A	50% MeOH, 100% MeOH and water		HPLC	India	[65]
Isorhamnetin	dried	0.03 mg/g	0.118 mg/g	Vacuum-drying	MeOH + HCl + ascorbic acid		HPLC	Taiwan	[71]
	freeze-dried	0.13 mg/g ^f^	0.52 mg/g ^f,g^	Freeze-drying	70% MeOH		HPLC-DAD-electrospray mass spectrometry	Ghana	[73]
		0.18 mg/g ^h^	0.72 mg/g ^g,h^						
Kaempferol	dried	0.04 mg/g	0.04 mg/g	Air-drying	MeOH + 1% *v*/*v* HCl + TBHQ		HPLC	Pakistan	[74]
	dried	ND	ND	N/A	50% MeOH		HPLC and MS/MS	India	[64]
	dried	2.360 mg/g	2.360 mg/g	N/A	50% MeOH, 100% MeOH and water		HPLC	India	[65]
	dried	0.198 mg/g	0.198 mg/g	N/A	Water at 80°C for 2 h		HPLC and MS/MS	India	[66]
	dried	0.36 mg/g	1.412 mg/g	Vacuum-drying	MeOH + HCl + 10 mg ascorbic acid		HPLC	Taiwan	[71]
	dried	0.8 mg/g	0.8 mg/g	Air-drying	70% MeOH + 0.1% acetic acid		LC/MS	Ghana	[72]
		1.23 mg/g	1.23 mg/g					Senegal	
		4.59 mg/g	4.59 mg/g					Zambia	
	freeze-dried	0.98 mg/g ^f^	3.92 mg/g ^f,g^	Freeze-drying	70% MeOH		HPLC-DAD-electrospray mass spectrometry	Ghana	[73]
		0.54 mg/g ^h^	2.16 mg/g ^g,h^						
	freeze-dried	2.25 mg/g	2.25 mg/g	Freeze-drying	80% MeOH		HPLC-DAD	Nicaragua	[58]
		1.75 mg/g	1.75 mg/g					India	
		1.05 mg/g	1.05 mg/g					Niger	
	freeze-dried	2.9 mg/g ^d^	2.9 mg/g ^d^	Freeze-drying	70% MeOH		LC/MS	Malawi	[75]
		2.3 mg/g	2.3 mg/g					Senegal	
		3.5 mg/g	3.5 mg/g					Nicaragua	
		0.3 mg/g ^c^	0.3 mg/g ^c^					ECHO	
		0.16 mg/g ^d^	0.16 mg/g ^d^					ECHO	
Luteolin	dried	ND	ND	N/A	MeOH + HCl + ascorbic acid		HPLC	Taiwan	[71]
Myricetin	dried	5.804 mg/g	5.804 mg/g	Air-drying	MeOH + 1% *v/v* HCl + TBHQ		HPLC	Pakistan	[74]
Quercetin	dried	0.281 mg/g	0.281 mg/g	Air-drying	MeOH + 1% *v/v* HCl + TBHQ		HPLC	Pakistan	[74]
	dried	0.207 mg/g	0.207 mg/g	N/A	50% MeOH		HPLC and MS/MS	India	[64]
	dried	0.207 mg/g	0.207 mg/g	N/A	50% MeOH, 100% MeOH and water		HPLC	India	[65]
	dried	0.807 mg/g	0.807 mg/g	N/A	Water at 80 °C for 2 h		HPLC and MS/MS	India	[66]
	dried	0.90 mg/g	3.529 mg/g	Vacuum-drying	MeOH + HCl + 10 mg ascorbic acid		HPLC	Taiwan	[71]
	dried	5.2 mg/g	5.2 mg/g	Air-drying	70% MeOH + 0.1% acetic acid		LC/MS	Ghana	[72]
		5.8 mg/g	5.8 mg/g					Senegal	
		7.57 mg/g	7.57 mg/g					Zambia	
	freeze-dried	3.21 mg/g ^f^	12.84 mg/g ^f,g^	Freeze-drying	70% MeOH		HPLC-DAD-electrospray mass spectrometry	Ghana	[73]
		4.16 mg/g ^h^	16.64 mg/g ^g,h^						
	freeze-dried	9.26 mg/g	9.26 mg/g	Freeze-drying	80% MeOH		HPLC-DAD	Nicaragua	[58]
		6.34 mg/g	6.34 mg/g					India	
		7.70 mg/g	7.70 mg/g					Niger	
	freeze-dried	5.47 mg/g ^b^	5.47 mg/g ^b^	Freeze-drying	70% MeOH		LC/MS	Malawi	[75]
		9.1 mg/g	9.1 mg/g					Senegal	
		15.2 mg/g	15.2 mg/g					Nicaragua	
		0.58 mg/g ^c^	0.58 mg/g ^c^					ECHO	
		0.46 mg/g ^d^	0.46 mg/g ^d^					ECHO	
Rutin	dried	0.390 mg/g	0.390 mg/g	N/A	50% MeOH, 100% MeOH and water		HPLC	India	[65]
	dried	ND	ND	N/A	50% MeOH		HPLC and MS/MS	India	[64]
	freeze-dried	1.674 mg/g	1.674 mg/g	Freeze-drying	80% EtOH		HPLC	Florida, USA	[57]

Abbreviations: ^a^ Obtained from the sum of single flavonoids measured; ^b^ Mean value of different samples; ^c^ Mature/old leaves; ^d^ Tender/young leaves; ^e^ Mean value of samples collected in different seasons; ^f^ Vegetative plants; ^g^ Obtained considering a moisture of 75%; ^h^ Flowering plants; ND = Not detected; N/A = Not available; QE = Quercetin equivalent; ECE = Epicatechine equivalent; CE = Catechin equivalent; IQE = Isoquercetin equivalent; RE = Rutein equivalent.

**Table 6:** Glucosinolates content in *Moringa oleifera* leaves.

Bioactive Compound	Leaves	Value Found in Literature	Value Express as Dry Weight	Drying Method	Extractive Method	Analytical Method	Country	Reference
**Glucosinolates**								
Benzyl	freeze-dried	ND ^a^ ND ^b^	ND ^a^ ND ^b^	Freeze-drying	70% MeOH	HPLC-DAD-electrospray mass spectrometry	Ghana	[73]
	freeze-dried	ND ^c^ ND ^d^	ND ^c^ ND ^d^	Freeze-drying	70% MeOH	LC/MS	Many countries	[75]
4-hydroxybenzyl (sinalbin)	freeze-dried	0.59 mg/g ^a^ ND ^b^	2.36 mg/g ^a,e^ ND ^b,e^	Freeze-drying	70% MeOH	HPLC-DAD-electrospray mass spectrometry	Ghana	[73]
4-(α-L-rhamnopyranosyloxy)-benzyl	freeze-dried	5.64 mg/g ^a^	22.56 mg/g ^a,e^	Freeze-drying	70% MeOH	HPLC-DAD-electrospray mass spectrometry	Ghana	[73]
		5.46 mg/g ^b^	21.84 mg/g ^b,e^					
	freeze-dried	33.9 mg/g ^c^	33.9 mg/g ^c^	Freeze-drying	70% MeOH	LC/MS	Many countries	[75]
		59.4 mg/g ^d^	59.4 mg/g ^d^					
4-O-(α-L-acetylrhamnopyranosyloxy)-benzyl isomer 1	freeze-dried	0.69 mg/g ^a^	2.76 mg/g ^a,e^	Freeze-drying	70% MeOH	HPLC-DAD-electrospray mass spectrometry	Ghana	[73]
		0.54 mg/g ^b^	2.16 mg/g ^b,e^					
	freeze-dried	2.9 mg/g ^c^	2.9 mg/g ^c^	Freeze-drying	70% MeOH	LC/MS	Many countries	[75]
		5.0 mg/g ^d^	5.0 mg/g ^d^					
4-O-(α-L-acetylrhamnopyranosyloxy)-benzyl isomer 2	freeze-dried	0.45 mg/g ^a^	1.80 mg/g ^a,e^	Freeze-drying	70% MeOH	HPLC-DAD-electrospray mass spectrometry	Ghana	[73]
		0.38 mg/g ^b^	1.52 mg/g ^b,e^					
	freeze-dried	1.2 mg/g ^c^	1.2 mg/g ^c^	Freeze-drying	70% MeOH	LC/MS	Many countries	[75]
		1.5 mg/g ^d^	1.5 mg/g ^d^					
4-O-(α-L-acetylrhamnopyranosyloxy)-benzyl isomer 3	freeze-dried	5.04 mg/g ^a^	20.16 mg/g ^a,e^	Freeze-drying	70% MeOH	HPLC-DAD-electrospray mass spectrometry	Ghana	[73]
		3.19 mg/g ^b^	12.76 mg/g ^b,e^					
	freeze-dried	17.4 mg/g ^c^	17.4 mg/g ^c^	Freeze-drying	70% MeOH	LC/MS	Many countries	[75]
		50.2 mg/g ^d^	50.2 mg/g ^d^					

Abbreviations: ^a^ Vegetative plants; ^b^ Flowering plants; ^c^ Mature/old leaves; ^d^ Tender/young leaves; ^e^ Obtained considering a moisture of 75%; ND = Not detected.

**Table 7:** Tannins content in *Moringa oleifera* leaves.

Bioactive Compound	Leaves	Value Found in Literature	Value Express as Dry Weight	Drying Method	Extractive Method	Analytical Method	Country	Reference
**Tannins**								
Total tannins	dried	13.2 gTAE/kg	13.2 gTAE/kg	Air-drying	Acetone/Water (7:3)	Folin-Ciocalteau modified	India	[68]
	dried	ND ^a^ 14.0 gTAE/kg ^b^	ND ^a^ 14.0 gTAE/kg ^b^	Air-drying	80% EtOH	Folin-Ciocalteau modified	Nicaragua	[69]
	dried	20.6 g/kg	20.6 g/kg	Air-drying at 35 °C for 24 h	Double lipid extraction with n-hexane (1:5)	N/A	Brazil	[76]
	freeze-dried	12 g/kg	12 g/kg	Freeze-drying	80% MeOH	Folin-Ciocalteau modified	Nicaragua	[77]
	freeze-dried	5 g/kg	5 g/kg	Freeze-drying	80% EtOH	Folin-Ciocalteau modified	Niger	[78]
Condensed tannins	dried	1.05 gLE/kg	1.05 gLE/kg	Air-drying	Acetone/Water (7:3)	Butanol–HCl–iron method	India	[68]
	dried	3.12 g/kg	3.12 g/kg	Air-dried under shade	N/A	Butanol–HCl–iron method	South Africa	[61]

Abbreviations: ^a^ Extracted leaves; ^b^ Unextracted leaves; TAE = Tannin acid equivalent; LE = Leucocyanidin equivalent; ND = Not detected; N/A = Not available.

**Table 8:** Saponins content in *Moringa oleifera* leaves.

Bioactive Compound	Leaves	Value Found in Literature	Value Express as Dry Weight	Drying Method	Extractive Method	Analytical Method	Country	Reference
**Saponins**								
Total saponins	dried	2.0 gDE/kg ^a^	2.0 gDE/kg ^a^	Air-drying	80% EtOH	Spectrophotometric method	Nicaragua	[69]
		50.0 gDE/kg ^b^	50.0 gDE/kg ^b^					
	freeze-dried	81 gDE/kg	81 gDE/kg	Freeze-drying	80% MeOH	Spectrophotometric method	Nicaragua	[77]
	freeze-dried	64 gDE/kg	64 gDE/kg	Freeze-drying	80% EtOH	Spectrophotometric method	Niger	[78]

Abbreviations: ^a^ Extracted leaves; ^b^ Unextracted leaves; DE = Diosgenin equivalent.

**Table 9:** Oxalates and phytates content in *Moringa oleifera* leaves.

Bioactive Compound	Leaves	Value Found in Literature	Value Express as Dry Weight	Drying Method	Extractive Method	Analytical Method	Country	Reference
**Oxalates and phytates**								
Oxalates	dried	430 mg/100 g	430 mg/100 g	Sun-drying for 4 days	N/A	AOAC 2004	India	[55]
		500 mg/100 g	500 mg/100 g	Shadow-drying for 6 days				
		450 mg/100 g	450 mg/100 g	Oven-drying at 60 °C for 1 h				
	dried	1050 mg/100 g	1050 mg/100 g	Air-drying at 35 °C for 24 h	Double lipid extraction with n-hexane (1:5)	N/A	Brazil	[76]
Phytates	dried	25.0 g/kg ^a^	25.0 g/kg ^a^	Air-drying	3.5% HCl for 1 h	Colorimetric method	Nicaragua	[69]
		31.0 g/kg ^b^	31.0 g/kg ^b^					
	freeze-dried	21.0 g/kg	21.0 g/kg	Freeze-drying	3.5% HCl for 1 h	Colorimetric method	Nicaragua	[77]
	freeze-dried	23.0 g/kg	23.0 g/kg	Freeze-drying	3.5% HCl for 1 h	Colorimetric method	Niger	[78]

Abbreviations: ^a^ Extracted leaves; ^b^ Unextracted leaves; N/A = Not available.

### 6.1. Vitamins

Fresh leaves of *Moringa oleifera* are reported to contain 11,300–23,000 IU of vitamin A [14,52]. Vitamin A plays key roles in many physiological processes such as vision, reproduction, embryonic growth and development, immune competence, cell differentiation, cell proliferation and apoptosis, maintenance of epithelial tissue, and brain function. Its deficiency is still prevalent in many developing countries, and considered responsible for child and maternal mortality [79].

Fresh leaves of *Moringa oleifara* are also a good source of carotenoids with pro-vitamin A action. They contain 6.6–6.8 mg/100 g [53,62] of β-carotene, greater that carrots, pumpkin and apricots (6.9, 3.6 and 2.2 mg/100 g, respectively) [80].

β-carotene is more concentrated in the dried leaves, with amounts ranging from 17.6 to 39.6 mg/100 g of dry weight (DW) [53,55,61]. This wide range may be explained by the different environmental conditions existing among different origin countries, genetic of the plant, drying method [55] and the different extraction and analysis methods employed as well. Freeze-drying seems to be the most conservative dehydration method. In freeze-drying leaves the β-carotene content is approximately 66 mg/100 g [57].

*Moringa oleifera* is an interesting source of vitamin C. Fresh leaves contain approximately 200 mg/100 g [14], greater than orange [80]. These amounts are of particular interest, as the vitamin C intervenes in the synthesis and metabolism of many compounds, like tyrosine, folic acid and tryptophan, hydroxylation of glycine, proline, lysine carnitine and catecholamine. It facilitates the conversion of cholesterol into bile acids and hence lowers blood cholesterol levels and increases the absorption of iron in the gut by reducing ferric to ferrous state. Finally, it acts as antioxidant, protecting the body from various deleterious effects of free radicals, pollutants and toxins [81]. However, being vitamin C sensitive to heat and oxygen, it is rapidly oxidized, so much so that its concentration in the *Moringa oleifera* dried leaves is lower than in the fresh leaves, dropping to 18.7 to 140 mg/100 g of DW [53,55,56].

Difference in (i) environmental conditions in the various origin countries; (ii) genetic of the plant; (iii) drying method [55] and (iv) different extraction and analysis methods, may explain the wide range of vitamin C content in *Moringa* leaves reported in literature. Freeze-drying seem to better preserve vitamin C from oxidation, so much so that greater amounts of this vitamin were found in leaves undergone to freeze-drying soon after the collection. In these latter, vitamin C concentration ranges between 271 and 920 mg/100 g of DW [57,58].

*Moringa oleifera* fresh leaves are a good source of vitamin E (in particular α-tocopherol) and contain approximately 9.0 mg/100 g [59] of this compound, similarly to nuts [80]. Vitamin E acts mainly as liposoluble antioxidants, but it is also involved in the modulation of gene expression, inhibition of cell proliferation, platelet aggregation, monocyte adhesion and regulation of bone mass [82]. Drying procedure determines a concentration of vitamin E up to values of 74.45–122.16 mg/100 g of DW [53,60,61].

Among vitamins of group B, only thiamine, riboflavin and niacin seem present in *Moringa oleifera* leaves. These vitamins mainly act as cofactors of many enzymes involved in the metabolism of nutrients and energy production, and their concentration in fresh leaves ranges between 0.06 and 0.6 mg/100 g, 0.05 and 0.17 mg/100 g and 0.8 and 0.82 mg/100 g for thiamine, riboflavin and niacin, respectively [14,53,60], similarly to fruits and vegetable [80]. Only one study reported the contribution of vitamin B1, B2 and B3 of dried leaves of *Moringa oleifera* [53]. Their concentrations were 2.85, 22.16 and 8.86 mg/100g of DW, respectively. However, the amount of riboflavin in dried leaves seems very high compared to that of fresh leaves. Further studies are needed to confirm these values. Finally, Girija *et al.* showed an appreciable physiological availability of these three vitamins in leaves of *Moringa oleifera* (61.6%, 51.5% and 39.9%, respectively) [54].

We did not find studies about other vitamin of group B or vitamin D and K in *Moringa oleifera* leave; therefore further studies on this topic are needed.

### 6.2. Polyphenols

*Moringa oleifera* dried leaves are a great source of polyphenols. Their concentrations range from 2090 to 12,200 mgGAE/100 g of DW [63,64,65,66,67] (or 1600 to 3400 mgTAE/100g of DW) [68,69]. These amounts are greater than those found in fruits and vegetable [83,84,85]. The different environmental conditions in the various origin countries, the harvesting season [56], the genetic of the plant, the drying method, the leaf maturity stage [63] and the extractive method used [67] may explain such wide range of reported values. Principal polyphenol compounds in *Moringa oleifera* leaves are flavonoids and phenolic acids.

### 6.3. Flavonoids

Flavonoids are a sub-group of polyphenolic compounds having a benzo-γ-pyrone structure and are ubiquitous in plants, as they are synthesized in response to microbial infections [86]. Epidemiological studies have consistently shown that high intake of flavonoids has protective effects against many infectious (bacterial and viral diseases) and degenerative diseases such as cardiovascular diseases, cancers, and other age-related diseases [86,87]. *Moringa oleifera* leaves are an interesting source of flavonoids compounds. Total flavonoids concentration in dried leaves ranges from 5.059 to 12.16 mg/g of DW [71], namely, close to or larger than that in many fruits and vegetable normally consumed [72,88]. These values are indeed the overall sum of the amounts of single flavonoids. However, some flavonoids were studied only by some authors and, therefore, these amounts may be inaccurate. The total concentration of flavonoids in freeze-dried leaves ranges from 21.0 to 61.62 mgRE/g of DW [57,58]. Myricetin, quercetin and kaempferol are the main flavonoids found in *Moringa oleifera* leaves. In dried leaves, myricetin concentration is approximately 5.804 mg/g of DW, while quercetin and kaempferol concentrations range from 0.207 to 7.57 mg/g of DW and not detectable amounts (ND) to 4.59 mg/g of DW, respectively [64,65,66,71,72,74]. Higher amounts were found in freeze-dried leaves. In particular, quercitin and kaempferol concentrations range from 5.47 to 16.64 mg/g and 1.5 to 3.5 mg/g of DW, respectively [58,73,75]. Isorhamnetin concentration in dried leaves is approximately 0.118 mg/g of DW [71], while, in freeze-dried leaves, its concentration is up to 7 times larger, ranging from 0.52 to 0.72 mg/g of DW [73]. Other flavonoids, such as luteolin, apigenin, daidzein and genistein, were found in not detectable concentrations in *Moringa oleifera* leaves [65,71]. However these compounds were investigated only in few studies and, therefore, further investigations are needed. In addition, in this case, the high inter-studies variations for these compounds may be explained taking into account different environmental conditions, harvesting season, genetic of the plant, drying method, leaf maturity stage, extraction method used and, finally, the different sensitivity of the analytical methods.

### 6.4. Phenolic Acids

Phenolic acids are a sub-group of phenolic compounds derived from hydroxybenzoic acid and hydroxycinnamic acid, naturally present in plants. Thanks to their documented effects on human health, the contribution of food-supplied phenolic acids is a subject of increasing interest. In particular, these compounds are mainly studied for their documented antioxidant, anti-inflammatory, antimutagenic and anticancer properties [89,90,91,92]. Particularly abundant in fruit and vegetables, phenolic acids were found in great amounts in *Moringa oleifera* leaves too. In dried leaves, gallic acid seems to be the most abundant, with a concentration of approximately 1.034 mg/g of DW [64,66], although Bajpai *et al.* [65] only found poorly detectable amounts. The concentration of chlorogenic and caffeic acids ranges from 0.018 to 0.489 mg/g of DW and ND to 0.409 mg/g of DW, respectively [64,65,66]. Lower, but appreciable, concentrations were found for ellagic and ferulic acids. Their concentrations range from ND to 0.189 mg/g and 0.078 to 0.128 mg/g of DW, respectively [64,65,66]. Some of these compounds were found more concentrated in freeze-dried leaves. Specifically, Zhang *et al.* [57], in leaves harvested in Florida and subsequently freeze-dried, found approximately 6.457 mg/g of DW of o-coumaric acid and 0.536 mg/g of DW of caffeic acid, while p-coumaric, synaptic, gentistic and syringic acids were found in poorly detectable amounts [57]. Like for the flavonoids, the different environmental conditions, harvesting season, genetic of the plant, drying method, leaf maturity stage, extraction method used and the different sensitivity of the analytical methods may have contributed to the high inter-study variation in the concentrations of phenolic acids in *Moringa oleifera* leaves.

### 6.5. Alkaloids

Alkaloids are a group of naturally occurring chemical compounds that contain mostly basic nitrogen atoms. This nitrogen may occur in the form of a primary amine (RNH_2_), a secondary amine (R_2_NH) or a tertiary amine (R_3_N). In addition to carbon, hydrogen and nitrogen, most alkaloids contain oxygen [93]. Alkaloids are of particular interest thanks to their pharmacological properties. The presence of these compounds has been confirmed in *Moringa oleifera* leaves [45,63]. Several of these compounds, such as *N*,α-l-rhamnopyranosyl vincosamide, 4-(α-l-rhamnopyranosyloxy) phenylacetonitrile (niazirin), pyrrolemarumine 4′′-*O*-α-l-rhamnopyranoside, 4′-hydroxy phenylethanamide-α-l-rhamnopyranoside (marumoside A) and its 3-*O*-β-d-glucopyranosyl-derivative (marumoside B) and methyl 4-(α-l-rhamnopyranosyloxy)-benzylcarbamate, have been isolated in *Moringa oleifera* leaves [94,95]. However, their amounts in the leaves are still unknown.

### 6.6. Glucosinolates and Isothiocyanates

Glucosinolates are a group of secondary metabolites in plants. Structurally they are β-*S*-glucosides of thio-oxime-*O*-sulfates and synthesized from amino acids. Appreciable amounts of these compounds were found in *Moringa oleifera* leaves. In particular, around 116 and 63 mg/g of DW in young and older leaves, respectively, are reported [75,96]. These amounts are close to, and in some case larger than, those found in many cruciferous vegetables (e.g., broccoli, cabbage, radish), mainly sources of these compounds [97]. 4-*O*-(α-l-rhamnopyranosyloxy)-benzyl glucosinolate has been identified as the dominant leaf glucosinolate of *Moringa oleifera* and is accompanied by lower levels of three isomeric 4-*O*-(α-l-acetylrhamnopyranosyloxy)-benzyl glucosinolates, which reflect the three position of the acetyl group at the rhamnose moiety of the molecule [75,96]. The concentrations of these compounds seem affected by the physiological stage of the plant and by the maturity stage of the leaves. The concentration of 4-*O*-(α-l-rhamnopyranosyloxy)-benzyl glucosinolate ranges from 21.84 to 59.4 mg/g of DW, while the concentrations of the three isomer of 4-*O*-(α-l-acetylrhamnopyranosyloxy)-benzyl glucosinolates range from 2.16 to 5.0 mg/g of DW, 1.2 to 1.8 mg/g of DW and 12.76 to 50.2 mg/g of DW for isomer 1, 2 and 3, respectively [73,75]. Amaglo *et al.* [73] report the presence of 4-hydroxybenzyl (sinalbin), with a concentration ranging between ND and 2.36 mg/g of DW. Glucosinolates can be hydrolyzed by myrosinase to produce d-glucose and various other degradation products like isothiocyanates [98], which are also present in *Moringa oleifera* leaves [99,100]. Both glucosinolates and isothiocyanates play an important role in health promoting and prevention of disease [101].

### 6.7. Tannins

Tannins are water-soluble phenolic compounds that bind to and precipitate alkaloids, gelatin and other proteins. They exhibit various biological properties: anti-cancer, antiatherosclerotic, anti-inflammatory, anti-hepatoxic, antibacterial and anti-HIV replication activity [102]. *Moringa oleifera* leaves are an appreciable source of tannins. Their concentrations range between 13.2 and 20.6 gTAE/kg [68,69,76] in dried leaves and between 5.0 and 12.0 gTAE/kg in freeze-dried leaves [77,78]. These amounts are greater than concentrations found in nuts [103], similar to those found in some plants [104] and berries [105], but much lower compared to the concenctrations found in other medicinal plants [106].

### 6.8. Saponins

Saponins are a group of natural compounds that consist of an isoprenoidal-derived aglycone, designated genin or sapogenin, covalently linked to one or more sugar moieties [107]. Even though some saponins have hemolytic side effects, they are studied for their anti-cancer properties [108,109]. Moringa oleifera leaves are a good source of saponins. Their concentration in dried leaves is approximately 50 gDE/kg of DW [69], while in freeze-dried leaves it ranges between 64 and 81 gDE/kg of DW [77,78]. These amounts are greater than the concentrations found in other plants [106], but slighty lower than ginseng root [110], one of the mainly source of these compounds.

### 6.9. Oxalates and Phytates

Oxalates and phytates are anti-nutritional compounds as they bind minerals inhibiting the intestinal absorption. Moringa oleifera leaves present high contents of these compunds. Oxalates content of dried leaves range from 430 to 1050 mg/100 g of DW [55,76], similar to other plants rich in these compounds [111], while phytates concentration range from 25 to 31 g/kg of DW [69] in dried leaves and from 21 and 23 g/kg of DW in freeze-dried leaves [77,78]. These amounts are greater than those found in legumes and cereals [112,113], but lower than brans [112].

## 7. Pharmacology

### 7.1. Antioxidant Properties

*Moringa oleifera* leaves are a rich source of antioxidant compounds [114].

Many *in vitro* studies on antioxidant activity of Moringa oleifera leaves are available in literature [56,58,63,64,65,67,70,115,116,117,118,119]. Siddhuraju and Becker [58] examined the radical scavenging capacities and antioxidant activities of the aqueous, aqueous methanol, and aqueous ethanol extracts of freeze-dried leaves of *Moringa oleifera* from different agro-climatic regions. The authors found that different leaves extracts inhibited 89.7%–92.0% peroxidation of linoleic acid and had a scavenging activity on superoxide radicals in a dose-dependent manner (EC_50_ within the range of 0.08–0.2 mg/mL, with the exception of water extract from Indian leaves which has an EC_50_ > 0.3 mg/mL). All of the solvent extracts of leaf samples had a very high radical scavenging activity, however better results were obtained in methanol and ethanol extracts. Both methanol and ethanol extracts of Indian origins showed the highest antioxidant activities, 65.1% and 66.8%, respectively, in the β-carotene-linoleic acid system. Nonetheless, increasing concentration of all the extracts had significantly increased reducing power (FRAP). The authors concluded that both methanol (80%) and ethanol (70%) were the best solvents for the extraction of antioxidant compounds from *Moringa oleifera* leaves. Similar results were obtained by Vongsak *et al.* [70] who measured antioxidant activity using various extraction methods on Thai *Moringa oleifera* leaves. The authors found that the extract obtained macerating dried leaves with 70% ethanol exhibited high DPPH-scavenging activity (EC_50_ = 62.94 g/mL) and the highest FRAP value (51.50 mmol FeSO_4_ equivalents/100 g extract). Iqbal and Bhanger [56] showed that season and agroclimatic locations have profound effect on the antioxidant activity of *Moringa oleifera* leaves. In this study, the authors collected the leaves in the months of December, March, June, and September from five different areas of Pakistan, and found that the overall antioxidant efficacy was greater in December or March depending upon location, and least in June. The authors suggested that the environmental temperature and, most likely, the soil properties have significant effects on antioxidant activity of *Moringa oleifera* leaves. The effect of season on antioxidant activity of *Moringa oleifera* leaves was also found by Shih *et al.* [115] in leaves collected in Taiwan. The results of this study showed that the winter samples (DPPH-radical scavenging EC_50_ = 200 μg/mL) of *Moringa oleifera* leaves had a significant stronger antioxidant activity than summer samples (DPPH-radical scavenging EC_50_ = 387 μg/mL). Finally, Sreelatha and Padma [63] evaluated the antioxidant activity of leaves collected in India in two stages of maturity. The analysis revealed only minor, but significant; differences in the two maturity stages, mature and tender leaves, for the DPPH scavenging activity (EC_50_ = 18.15 *vs.* 19.12 μg/mL), superoxide scavenging activity (EC_50_ = 12.71 *vs.* 15.51 μg/mL), nitric oxide scavenging activity (EC_50_ = 56.77 *vs.* 65.88 μg/mL) and for lipid peroxidation inhibition (EC_50_ = 25.32 *vs.* 30.15 μg/mL).

Verma *et al.* [120] examinated the effects of administration of 50 and 100 mg/kg bw/day of ethyl acetate/polyphenolic extract of *Moringa oleifera* leaves for 14 days on markers of oxidative stress in mice treated with CCl_4_. The authors observed that the supplementation with *Moringa oleifera* leaves extract in CCl_4_-intoxicated rats prevented the increment of lipid peroxide oxidation (LPO) levels, the decrement of glutathione (GSH) concentration and in the activities of superoxide dismutase (SOD) and catalase (CAT) antioxidant enzymes in liver and kidney compared to negative control. Interestingly, the effects obtained in the group treated with 100 mg/kg bw/day of leaves extract were comparable to those obtained in the standard group treated with 50 mg/kg bw/day of vitamin E. Similarly, Luqman *et al.* [121] studied the effects of administration of increasing amounts of both aqueous and alcoholic extract of *Moringa oleifera* leaves on markers of oxidative stress in Swiss albino mice. The authors observed an enhancement of GSH concentration in mice erythrocytes treated with the aqueous extract of *Moringa oleifera* leaves. The observed rise is consistent with the increase in the dose of the extract. Similarly, the dose-responsive effect on LPO was also observed. Moreover, the basal value of malondialdehyde (MDA) concentration was maintained in case of the aqueous extract of *Moringa oleifera* leaves. Milder, but appreciable, results were obtained using ethanolic extract. Moyo *et al.* [122] studied the antioxidant properties of diet supplemented with *Moringa oleifera* dried leaves in goats model. The authors found a significant increment of the activity of GSH in goats supplemented with Moringa leaves as compared with the control group. In comparison, the activities of catalase (CAT) and superoxide dismutase (SOD) of diet supplemented with *Moringa oleifera* were increased appreciably than the goats fed with ordinary feed. Finally, the supplementation of *Moringa oleifera* leaves inhibited the amount of MDA generated in liver homogenate. Therefore, also the consumption of *Moringa oleifera* leaves could protect the animals against diseases induced by oxidative stress. Finally, the administration of *Moringa oleifera* leaves extract seems to prevent also the oxidative damages caused by high-fat diet. Das *et al.* [123] reported a lower MDA concentration, a higher GSH concentration and hepatic FRAP in mice fed with high-fat diet and supplemented with *Moringa oleifera* leaves extract compared to mice only treated with high-fat diet.

Only one study reported the effects of *Moringa oleifera* leaves extract on oxidative modification of LDL using an “*ex vivo*” assay [114]. In this study, human plasma was collected and preincubated at 37 °C for 1 h with various concentrations of freeze-dried *Moringa oleifera* leaves extract (1, 10, 30 and 50 µg/mL). Control group was treated with vitamin E as standard antioxidant. Oxidation reaction of LDL was initiated by adding freshly prepared 10 µM CuSO_4_ to the samples. This experiment showed that in the presence of *Moringa oleifera* leaves extract the oxidative modifications of human plasma LDL were significantly inhibited in a dose-dependent manner. In the presence of the leaves extract at a final concentration of 1, 10, 30, and 50 g/mL, the lag-time of conjugated diene formation was increased to 45.0, 151.66, 251.67, and 340.33 min, respectively with an IC_50_ at 2 h of 5 µg/mL. Moreover, in the presence of *Moringa oleifera* leaves extract at a final concentration of 1 and 10 g/mL, the thiobarbituric acid reactive substances (TBARS, products of lipid peroxidation) formation was reduced to 104.16 and 2.80 nmol/mg LDL protein, respectively. Interestingly, the incubation with higher concentrations of the leaf extract completely blocked TBARS formation. These findings indicate that *Moringa oleifera* leaves extract could suppress the initiation and propagation of lipid peroxidation also in humans.

In conclusion, many *in vitro* and *in vivo* studies have shown antioxidant properties of *Moringa oleifera* leaves. These findings may be explained by the abundant amounts of antioxidant compounds in the leaves. However further studies in human are needed to confirm the results obtained in animals.

### 7.2. Anti-Inflammatory and Immunomodulatory Properties

Inflammation is a protective immunovascular response that involves immune cells, blood vessels, and molecular mediators. The purpose of inflammation is to eliminate the initial cause of cell injury, clear out necrotic cells and tissues damaged from the original insult and the inflammatory process, and to initiate tissue repair.

The anti-inflammatory properties of *Moringa oleifera* seeds have been so far reported in a number of studies, while only few studies on anti-inflammatory effect of leaves are available in the literature.

Coppin *et al.* [72] found that 2 on 3 samples of *Moringa oleifera* leaves were able to inhibit nitric oxide (NO) production by macrophage cells after treatment with bacterial lipopolysaccharide (LPS), whereas the third sample was found not active. The authors attributed this difference to genetics and chemistry of plants which can significantly vary even within the same species. Kooltheat *et al.* [124] found that the ethyl acetate extract of *Moringa oleifera* leaves was able to inhibits human macrophage cytokine production (TNF-α, IL-6 and IL-8) induced by extract of cigarette smoke and by LPS, similar to Aspirin (reference drug). Finally, Waterman *et al.* [100] observed that both *Moringa oleifera* concentrate and isothiocyanates isolated from the leaves significantly decreased gene expression and production of inflammatory markers in RAW macrophages. Specifically, both attenuated expression of inducible nitric oxide synthase (iNOS), IL-1β and production of NO and TNFα at 1 and 5 μM.

Sudha *et al.* [125] and Gupta *et al.* [126] evaluated the immunomodulatory effect of methanolic and ethanolic extracts of *Moringa oleifera* leaves, respectively, in cyclophosphamide-induced immunodeficient mice. These authors found that Moringa oleifera leaves extract shown a significant increase in white blood cells, percent neutrophils and serum immunoglobulins, suggesting that it stimulate both cellular and humoral immune response. These results were confirmed by other studies [127,128,129]. Rajanandh *et al.* [130] evaluated the anti-inflammatory effects of extract of *Moringa oleifera* leaves in rats fed with an atherogenic diet in order to induce hyperlipidemia. In this study, rats were fed with 100 and 200 mg/kg bw of hydro alcoholic extract of *Moringa oleifera* leaves for a period of 28 days. At the end of the experiment, serum TNF-α and IL-1 were significantly lower in rats treated with both doses of hydro-alcoholic extract of *Moringa oleifera* leaves compared to control. Recently, Waterman *et al.* [99] evaluated the effect of supplementation of *Moringa oleifera* leaves concentrate in obese-induced mice with very high-fat diet (60% kcal from fat) for 12 weeks. In the experimental group, the very high-fat diet contained 5% of *Moringa oleifera* leaves concentrate. The authors observed a reduction of gene expression of pro-inflammatory markers, TNFα, IL-6 and IL-1β in the liver and ileum tissues in mice treated with *Moringa oleifera* concentrate compared to the control group. Similarly, in adipose tissue, gene expression of TNFα was reduced while adiponectin had enhanced expression in the treated mice compared to the controls. Finally, Das *et al.* [131] tested the anti-inflammatory effects of both *Moringa* oleifera leaves extract and quercitin in mice fed with high-fat diet. The authors found that both *Moringa* oleifera leaves extract and quercitin inhibited the translocation of p65 subunit of NF-κB (4.2- and 2.3-fold reduction in the expression level, respectively) in comparison with the group only fed with high-fat diet. Moreover, both *Moringa oleifera* leaves extract and quercitin down-regulated the expressions of iNOS, interferon gamma (IFN-γ) and C reactive protein (CRP) compared to the group only fed with high-fat diet. Finally, release of serum inflammatory cytokines TNF-a and IL-6 potently decreased in the group treated with the extract (30% and 27% reduction for TNF-a and IL-6, respectively) and in the group treated with quercitin (27% and 21% reduction for TNF-a and IL-6, respectively) in comparison to the HFD group.

In conclusion, many *in vitro* and *in vivo* studies in animal model have demonstrated the anti-inflammatory and immunodulatory effects of a supplementation with *Moringa oleifera* leaves. Many bioactive compounds may be involved in the anti-inflammatory process. Among them, quercetin seems to inhibit activation of NF-kB and also the subsequent NF-kB-dependent downstream events and inflammation [131]. However, many other bioactive compounds naturally present in *Moringa oleifera* leaves, such as other flavonoids and phenolic acids, may be involved in to anti-inflammatory process. Further studies should be devoted to investigate the potential anti-inflammatory action and the mechanism of action of other bioactive compound naturally present in *Moringa oleifera* leaves. Concerning immunomodulatory process, the exact mechanism of action of the extract of Moringa oleifera leaves in stimulation of both cellular and the humoral immunity is not clear yet [126]. Therefore, further studies on this subject are needed. Finally, human studies are needed to evaluate the anti-inflammatory and immunomudulatory properties of *Moringa oleifera* leaves also in human beings.

### 7.3. Hypoglycemic Properties

Hypoglycemic effects of Moringa oleifera leaves are reported in literature [132].

In the study of Ndong *et al.* [133], male spontaneously diabetic Goto-Kakizaki (GK) rats and non-diabetic male Wistar rats received a single dose of glucose solution and a dose *Moringa oleifera* leaves (2 g/kg BW and 200 mg/kg BW, respectively), whereas control groups of both animals only received a single dose of glucose solution. Blood glucose concentration was measured at 0, 10, 20, 30, 45, 60, 90 and 120 min. Results from OGTT shown that *Moringa oleifera* significantly decreased blood glucose at 20, 30, 45, and 60 min in GK rats compared to the control and at 10, 30 and 45 min Wistar rats compared to the control after glucose administration. Moreover, in GK rats, the treatment with *Moringa oleifera* leaves reduced AUC values by 23%, whereas it did not significantly affect these values in control rats. These results suggest that *Moringa oleifera* has a glucose intolerance ameliorating effect in both GK and Wistar rats, with a greater action in diabetic than in normoglycemic rats. Kar *et al.* [134] tested hypoglycaemic activity of ethanol extract (95%) of some Indian medicinal plants, including *Moringa oleifera*, in alloxan-induced diabetic rats. The authors found that a single dose of 250 mg/kg BW of leaves extract determined a halving of serum glucose in a week. In the study of Jaiswal *et al.* [135], the effect of the aqueous extract of *Moringa oleifera* leaves on glucose homeostasis was tested in healthy and streptozotocin-induced sub, mild and severely diabetic Wistar rats (STZ, a cytotoxic drug that selectively destroys islet β cells). The dose of 200 mg/kg BW of leaves extract determined a maximum fall of 26.7% in fasting blood glucose concentration and a maximum fall of 29.9% in OGGT at 3 h after glucose administration. The same dose determined a maximum fall of 31.1% and 32.8% in OGGT in sub and mild diabetic rats, respectively. Severely diabetic rat were, instead, long treated (21 days) with aqueous extract of *Moringa oleifera* leaves. The experiment revealed a fall of 25.9%, 53.5%, 69.2% in fasting blood glucose at 7, 14 and 21 days treatment with leaf extract. Interestingly, results obtained in sub, mild and severely diabetic rats were similar to those obtained in Glipizide treated rats (2.5 mg/kg BW, reference drug). Similar results were obtained by Edoga *et al.* [136]. In this study, the aqueous extract produced a dose-dependent reduction in blood glucose levels of normoglycemic and hyperglycemic rats. In normoglycemic rats, the aqueous extract of *Moringa oleifera* (100, 200 and 300 mg/kg) exhibited a reduction of 23.14%, 27.05% and 33.18% respectively of the blood glucose levels within 6 hours of administration, while in alloxan-induced diabetic rats the reduction were of 33.29%, 40.69% and 44.06% respectively. Interestingly, also in this study, similar results were obtained using 200 mg/kg of tolbutamide (reference drug). Divi *et al.* [137] tested the antidiabetic properties of aqueous extract of *Moringa oleifera* leaves in fructose-induced insulin-resistant (IR) and STZ-induced diabetic rats. After administration of 200 mg/kg BW of aqueous extract of *Moringa oleifera* leaves for 60 days the authors observed a decrease in blood glucose concentration in both groups and a decrease of insulin in IR group compared to respectively control. The hypoglycemic effect of aqueous extract of Moringa oleifera leaves in STZ-induced diabetic rats was also confirmed by Yassa *et al.* [138]. Moreover, in this study histopathological damage of islet cells was also markedly reversed. *Moringa oleifera* treatment significantly increased the areas of positive purple modified Gomori stained β-cells (from 60% to 91%) and decreased the area percentage of collagen fibers (from 199% to 120%) compared to control values. All these findings were confirmed by other studies using similar approach [139,140,141].

William *et al.* [142] examined the effects of *Moringa oleifera* leaves added to a standardized meal on serum post-prandial glucose concentration at 1 and 2 h from the consumption, compared to the standard meal alone or a 75 g oral glucose load in type 2 diabetes mellitus patients. Compared to the glucose load, standard meals with or without vegetable supplements induced a significantly lower post-prandial glucose response as derived from AUCs. However, leaf-supplemented meals caused a lower response (−21%) compared to standard meals alone. Moreover, plasma insulin AUCs did not differ significantly between the two meals, suggesting that the hypoglycemic effect of *Moringa oleifera* leaves supplementation was not due to increased insulin secretion. Kumari [143] examined the hypoglycemic effect of 40-days administration of *Moringa oleifera* leaves in non-insulin dependent type 2 diabetes mellitus subjects aged 30–60 years old. Recruited subjects were divided in experimental and control group: the first received 8 g of dried *Moringa oleifera* leaves for 40 days, whereas the control group didn’t receive any treatment. Daily meals were comparable between the two groups in terms of relative food type consumption, nutrients and calories as well. Fasting and post-prandial blood glucose concentrations were taken at baseline and at the end of the experiment. Fasting and post-prandial blood glucose did not differ much from baseline in the control group, while they were significantly reduced in the experimental group (−28% and −26%, respectively). Finally, Ghiridhari *et al.* [144] recruited a group of 60 normal weight type 2 diabetes mellitus patients, aged 40–58 years old, on sulfonylurea medication and a standardized calorie-restricted diet (1500 to 1800 Kcal). The patients were equally divided into an experimental and a control groups. Patients in the experimental group were prescribed an unspecified amount of *Moringa oleifera* leaf for 90 days. The results showed that post prandial blood glucose of experimental group initially was 210 mg/dL and it reduced to 191, 174 and 150 mg/dL respectively after the first, second and third month of supplementation (with a significant decrease of 9%, 17% and 29%, respectively). In control group post prandial blood glucose level of 179 mg/dL was substantially maintained during the whole experiment. Similar trends were observed for glycated hemoglobin (HbA 1c). In the experimental group initial value of 7.81% significantly decreased to 7.4% after the supplementation period, whereas it did not change in the control group. The results indicated that *Moringa oleifera* leaves are a suitable to reduce the diabetic complications in diabetic patients. However, it should be noted that treatment allocation to patients appear to have not been randomized as baseline values for the two parameters were higher in the experimental group than in the control group, 7.8% ± 0.5% *vs.* 7.4% ± 0.6% for HbA_1c_ and 210 ± 49 *vs.* 179 ± 36 mg/dL for post-prandial glucose response [132]. Finally, it has been suggested that isothicyanates isolated from *Moringa oleifera* leaves reduced glucose production in liver cells, showing activity at very low concentrations and being close to two orders of magnitude more active than metformin. These compounds were able to decrease phosphoenolpyruvate carboxykinase and glucose-6-phosphatase gene expression suggesting that they act via blocking these rate-limiting steps in liver gluconeogenesis [99].

In conclusion, scientific evidences suggest a potential use of *Moringa oleifera* leaves in the treatment of diabetes. Many compounds isolated in *Moringa oleifera* leaves may be involved in the glucose homeostasis. Among theme, isothiocyanates seem to reduce insulin resistance and hepatic gluconeogenesis [99]. However, also polyphenol compounds abundant in *Moringa oleifera* leaves, such as phenolic acids and flavonoids, may contribute to its effects on glucose homeostasis. These compounds exert, indeed, anti-diabetic effects targeting various cellular signaling pathways in pancreas, liver, skeletal muscle and white adipose tissue. In particular, they influence β-cell mass and function, as well as energy metabolism and insulin sensitivity in peripheral tissues. Their effects may be due to antioxidant, enzyme inhibition, receptor agonist or antagonist activity or through novel mechanisms yet to be elucidated [145,146,147,148,149]. Phenolic compounds, flavonoids and tannins may be also involved in the ability of Moringa oleifera leaves extract to inhibit the intestinal sucrase and, slightly, the pancreatic α-amylase actions [150]. Finally, even though studies on human being highlight the hypoglycemic effects of *Moringa oleifera* leaves, further larger randomized studies controlled for potential confounders, such as sex, age, race, nutritional status and dietary habits in human are required before using the leaves as herbal drug for the treatment of diabetes.

### 7.4. Hypolipidemic Properties

Hypolipidemic effects of *Moringa oleifera* leaves are reported in literature [132].

Chumark *et al.* [114] examined the hypolidipemic effects of *Moringa oleifera* leaves in rabbits fed with high-cholesterol diet (5%) for 12 weeks. A group was concomitantly treated with *Moringa oleifera* leaves extract (0.1 g/kg BW/day). At the end of the experiment, rabbits treated with *Moringa oleifera* leaves presented a total cholesterol, LDL, HDL and triglycerides significantly reduced of 52%, 42.7%, 44.2% and 75.4% respectively, compared to rabbits only fed with high-cholesterol diet, with consequent reduction of 86.52% of internal carotid atherosclerotic plaque formation. Similar trends were observed in the group treated with 5 mg/kg BW of simvastatin (reference drug). The anti-dyslipidemic effects of *Moringa oleifera* leaves were also examined in rats fed with a high-fat diet [151]. In this study, Wistar rats were fed for 30 days with a high-fat diet containing 20% (*w*/*w*) fat. Animals were divided in two groups, one of which received a daily dose of 1 g/kg BW of aqueous extract of *Moringa oleifera* leaves. The authors observed a significant lower increasing of serum cholesterol in treated compared to untreated rats, but not in liver and kidney. The percentage decrease in serum, liver and kidney was 14.35%, 6.40% and 11.09%, respectively. Similarly, Jain *et al.* [152], fed albino rats, for 30 days, with a high-fat diet containing 26% fat. In this case, three groups received different doses of methanolic extracts of *Moringa oleifera* leaves (150, 300, or 600 mg/kg bw/die), one group was treated with 4 mg/kg of simvastatin, whereas another group didn’t receive any supplementation. Treatment with methanolic extract, at three different doses significantly decreased the levels of total cholesterol and LDL as compare to the controls. The same was true for VLDL treating rats with the medium and the highest doses of methanolic extracts. Interestingly, the medium and the highest doses of methanolic extracts determined a significant increment of HDL. Similar results were obtained treating rats with simvastatin. In addition, Bais *et al.* [153] has recently shown that a supplementation of 200 and 400 mg/kg bw/die of *Moringa oleifera* leaves extract exhibited anti-obesity effects in high-fat fed mice. Lower food intake was observed in the groups treated with *Moringa oleifera* leaves extract when compared to the group feed with only high-fat diet. Subsequently, the body weight gain in these groups was significantly lower than the rats feed with only high-fat diet. All these findings were confirmed by other studies using similar approaches [130,153,154].

Other studies evaluated the hypolipidemic effects of *Moringa oleifera* leaves supplementation in diabetes-induced rats. Diabetes is indeed a risk condition to develop dyslipidemia. Oyedepo *et al.* [140] evaluated the effect of aqueous extract of *Moringa oleifera* leaves on plasma total cholesterol, triglycerides, HDL and LDL in alloxan-induced diabetic male rats. Diabetic rats were feed with 400 mg/kg BW of aqueous extract of *Moringa oleifera* leaves for 28 day. At the end of the experiment diabetic rats presented lower plasma total cholesterol, triglycerides and LDL, but not higher HDL, than diabetic rats untreated. Divi *et al.* [137] observed a decrease of triglycerides (60.1% and 56.4%), total cholesterol (28.8% and 18.0%), LDL (31.2% and 13.9%) and VLDL (60.4% and 56.5%) in both fructose-induced insulin resistant and stz-induced diabetic rats treated with *Moringa oleifera* leaves extract compared to respectively untreated control. Interestingly, an increment of HDL was observed only in diabetic rats (40.4%), but in IR rats in which a decrement (10.3%) was observed. Similar results were obtained by other studies [141].

Nambiar *et al.* [155] firstly examined the potential anti-dyslipidemic effect of *Moringa oleifera* leaves in 35 hyperlipidemic subjects. Subjects were divided control (18 subjects) and experimental (17 subjects) groups. Groups were similar for anthropometric values (age, height, weight, body mass index, waist/hip ratio) and their daily nutrient intake. The experimental group was treated with 4.4 g of dehydrated *Moringa oleifera* leaves, as four 550 mg tablets twice daily, for 50 days. Plasma lipid profiles were determined at the beginning and at the end of the experiment. The authors observed a significant decrease of 1.6% in plasma total cholesterol and an increment of 6.3% of HDL in experimental group compared to control group. Not significant trends were observed for LDL, VLDL, and triglycerides. However, the TC/HDL ratio significantly decreased by 6.6%, indicating that the treatment induced a lesser atherogenic lipid profile. Kumari [143] observed a significant decrease of total cholesterol (14%), LDL (29%), VLDL (15%) and triglycerides (14%) in diabetic subjects treated with 8 g of Moringa oleifera leaves for 40 days compared to untreated subjects. However, not significant increase of HDL was observed.

In conclusion, scientific evidences suggest a positive effect of *Moringa oleifera* leaves on lipid homeostasis. Many bioactive compounds may contribute to these effects. It has been suggested that phenolic compounds, in particular flavonoids, play important roles on lipid regulation [156]. Moreover, phenolic compounds of *Moringa oleifera* leaves extract seem to be involved in the inhibition of pancreatic cholesterol esterase activity reducing and delaying the cholesterol absorption, and binding bile acids by forming insoluble complexes and increasing their fecal excretion with theoretical decreasing of plasma cholesterol level [150]. However, studies on this topic are needed to confirm these hypotheses. Moreover, human are still few, and generally conducted on a restricted number of subjects. Therefore, further larger randomized studies controlled for potential confounders, such as sex, age, race, nutritional status and dietary habits in human are required before using the leaves as hypolipidemic and hypocholesterolemic herbal drug.

### 7.5. Hepato and Kidney Protective Properties

Controversial results about the effects of *Moringa oleifera* leaves on liver and kidney health are reported. Oyagbemi *et al.* [157] and Asiedu-Gyekye *et al.* [129] observed an increment in serum alanine aminotransferase (ALT), aspartate aminotransferase (AST), alkaline phosphatase (ALP), blood urea nitrose (BUN) and creatinine following an administration of the extract of *Moringa oleifera* leaves in mice. Being biomarkers of liver and kidney injury, the authors speculated that leaves might predispose to hepatic and kidney damage. However, histopathological examinations did not reveal any histological lesions in the sinusoids or central vein [129]. On the other hand, other studies [158,159,160,161] reported hepatic and kidney protective properties against several drugs, such as isoniazid, rifampicin, pyrazinamide, acetaminophen and gentamicin, attributable to *Moringa oleifera* leaves. The authors observed a reduction of serum ALT, AST, ALP [158,159,160] and BUN and creatinine [161] in animals treated with the extract of *Moringa oleifera* leaves. These findings were confirmed by histological examinations, which revealed an amelioration of the hepatic and kidney damages induced by drugs, in animals treated with *Moringa oleifera* leaves. Similar results were obtained by Adeyemi and Elebiyo [162] in rats co-treated with *Moringa oleifera* leaves and NiSO_4_ in order to induce nephrotoxicity. Finally, Das *et al.* [123] observed a reduction of ALT, AST and ALP and a lower liver damage in rats fed with high fat diet and co-treated with *Moringa oleifera* leaves, suggesting a potential role of the leaves in the prevention of nonalcoholic fatty liver disease (NAFLD).

In conclusion, scientific evidences suggest a potential role of Moringa oleifera leaves in the amelioration of the hepatic and kidney damages induced by drugs in animals. However, further studies on human beings are required before using Moringa as herbal medication.

### 7.6. Anticancer Properties

Experimental evidences showed the capacity of *Moringa oleifera* leaves to protect organism and cell from oxidative DNA damage associated with cancer and degenerative diseases [163,164].

Many *in Vitro* studies evaluated the anticancer properties of both water and alcoholic extracts of *Moringa oleifera* leaves on different types of tumor cells lines. Sreelatha *et al.* [165] found that the aqueous extract of *Moringa oleifera* leaves exhibited a dose-dependent inhibition of cell proliferation of KB human tumor (KB) cells line. This antiproliferative effect was also associated with an induction of apoptosis, morphological changes and DNA fragmentation. Tiloke *et al.* [166] observed a significant increment in reactive oxygen species (ROS) with a concomitant decrease in intracellular GSH levels caused by a reduction in Nrf2 protein (1.89-fold) and mRNA expression (1.44-fold) in human lung cancer cells treated with *Moringa oleifera* leaves extract compared to untreated cells. These oxidants can react with DNA in the cell determining a DNA fragmentation with consequent death of cell itself. The pro-apoptotic properties of *Moringa oleifera* leaves extract were also confirmed by the significant increase in p53 protein (1.02-fold) and mRNA expression (1.59-fold), in caspase-9 (1.28-fold) and caspase-3/7 (1.52-fold) activities and an enhanced expression of Smac/DIABLO in cells treated with the extract. *Moringa oleifera* leaves extract also caused the cleavage and activation of PARP-1 into 89 and 24 KDa fragments [166]. The apoptosis induction and tumor cell growth inhibition activities of aqueous extract of *Moringa oleifera* leaves on human lung cancer cells were also studied by Jung [167]. This study confirmed previous results and found that *Moringa oleifera* leaves extract showed greater cytotoxicity for tumor cells than for normal cells, strongly suggesting that it could be an ideal anticancer therapeutic candidate specific to cancer cells. In the study of Berkovich *et al.* [168], concentration ≥0.75 mg/mL of *Moringa oleifera* leaves extract determined a significant inhibition of pancreatic cancer cells (Panc-1) survival as a result of progressive cell apoptosis. In particular, the treatment with 2 mg/mL *Moringa oleifera* leaves extract resulted in a reduction of 98% of Panc-1 cells survival, attributable, at least in part, by a down-regulation of the expression of key NF-κB signaling pathway proteins. Parvathy and Umamaheshwari [169] found that methanolic extract of *Moringa oleifera* leaves exhibited less viability on myeloma cells both at highest dose (2% at 200 μg/mL) and at lowest dose (12% at 0.5 μg/mL). Khalafalla *et al.* [170] found that both hot water and ethanolic *Moringa oleifera* leaves extracts inhibited the viability of acute myeloid leukemia, acute lymphoblastic leukemia and hepatocellular carcinoma cells. On the other hand, all the tested extracts did not exhibit toxic effects against normal mononuclear cells. Charoensin [171] found that dichloromethane extract of *Moringa oleifera* leaves was more cytotoxic against human hepatocellular carcinoma (HepG2; IC_50_ = 120.37 µg/mL), colorectal adenocarcinoma (Caco-2; IC_50_ = 112.46 µg/mL) and breast adenocarcinoma (MCF-7; IC_50_ = 133.58 µg/mL) than methanolic extract (IC_50_ > 250 µg/mL). Both extracts had no toxicity on human fibroblast. Finally, Pamok *et al.* [172] observed that both aqueous and ethanolic extract inhibited cell proliferation of three different types of colon cancer cells lines, with better results using ethanolic extract.

Only one *in vivo* study is available in literature about anticancer properties of the extract of *Moringa oleifera* leaves. Purwal *et al.* [173] studied the effects of oral administration of hydromethanolic and methanolic extracts of Moringa oleifera leaves in murine melanoma tumor model. The authors observed that an oral administration of 500 mg/kg for 15 days of the extracts determined a delay in the growth of tumors and a significant increase of life span of 48% and 32% for methanolic and hydrometanolic extracts respectively.

In conclusion, *in vitro* studies suggest potential anti-cancer properties of *Moringa oleifera* leaves. These properties may be explained by the presence of several bioactive compounds, such as 4-(α-l-rhamnosyloxy) benzyl isothiocyanate, niazimicin and β-sitosterol-3-*O*-β-d-glucopyranoside [174,175]. However, further animal studies are needed to confirm these effects. Finally, no studies on human are available in literature.

## 8. Conclusions

*Moringa oleifera* is one of the most studied and used plants. Its uses stretch from food and medicinal uses to water purification, biopesticide and production of biodiesel.

Moringa shows diversifications in many features and high morphological variability which may become a resource for the conservation and the selection of *Moringa oleifera* germplasm. However, some questions still have to be addressed, *i.e.*, collection and characterization of world accessions (both cultivated and natural) and setting a collaborative network among all institutions that already work on *Moringa oleifera*. This will help scientists and producers to have reliable access to information and materials and better develop Moringa. Moreover, researches focused on the association between phenotypical and molecular data and on genetic maps (both association map and physical map) in order to identify genes are needed within the contest of breeding. Next generation sequencing (NGS) could be an approachable tool to discover genome-wide genetic markers and building saturated genetic map within reasonable cost and time.

Nevertheless *Moringa oleifera* is an interesting plant for its contribution in bioactive compounds. In particular, leaves, the most used part of the plant, are rich in vitamins, carotenoids, polyphenol, phenolic acids, flavonoids, alkaloids, glucosinolates, isothiocyanates, tannins and saponins. In addition, even if leaves present high variation in the amounts of bioactive compounds as a result of the genetic characteristics of the plant, the environmental conditions to which the plant is subjected and the post-harvest treatments as well, they present greater amounts of these compounds than fruits, vegetables and other plants generally used in human nutrition. On the other hand, the high leaves content of oxalates and phytates could limit the intestinal adsorption of minerals. Therefore, this aspect should be taken in to account for future nutritional researches focused on using Moringa as minerals supplementation.

The high contribution in bioactive compounds may explain the pharmacological properties ascribed to *Moringa oleifera* leaves. Many *in vitro* and *in vivo* studies in animals have widely confirmed numerous pharmacological properties. However, few evidences on human beings are available. Therefore, it is too early to recommend *Moringa oleifera* leaves as medication in the prevention or treatment of diabetes, cardiovascular disease, dyslipidemia, cancer and infective diseases. Further studies aimed to confirm the pharmacological effects of moringa on human beings and, at the same time, ensuring its safety on human health consequently to a chronic or long-term use should be encouraged.
